# Isolation and *in vitro* assessment of chicken gut microbes for probiotic potential

**DOI:** 10.3389/fmicb.2024.1278439

**Published:** 2024-01-29

**Authors:** Fatima Shahbaz, Fatima Muccee, Aansa Shahab, Sher Zaman Safi, Suliman Yousef Alomar, Abdul Qadeer

**Affiliations:** ^1^School of Biochemistry and Biotechnology, University of the Punjab, Lahore, Pakistan; ^2^Faculty of Medicine, MAHSA University, Kuala Lumpur, Malaysia; ^3^Department of Zoology, College of Science, King Saud University, Riyadh, Saudi Arabia; ^4^Department of Cell Biology, School of Life Sciences, Central South University, Changsha, China

**Keywords:** probiotics, bile salts, gut, chicken, poultry, hemolysis, adhesion

## Abstract

Poultry production occupies an important place in the economy of any country. High broiler production in recent years has badly affected its profitability due to bad feed quality, excessive use of chemotherapeutic agents, emergence of diverse pathogens, and the deficiencies in management practices during rearing cycle. Microbiological improvement of the meat quality using potential probiotics can be beneficial for broiler farming. Present study was initiated to isolate chicken gastrointestinal tract (GIT) bacteria with probiotic potential. To isolate probiotics from chicken gut, alimentary canal of chickens of known sizes and ages was suspended in ringers soln. Under shaking conditions for overnight followed by serial dilutions of ringers soln. Bacterial isolates were analyzed via growth curve analysis, biochemical testing using RapID™ NF Plus Panel kit, molecular characterization, antimicrobial activity assay, antibiotic sensitivity assay, GIT adherence assay, bile salt and gastric acid resistant assay, and cholesterol assimilation assay. Four bacteria isolated in present study were identified as *Limosilactobacillus antri* strain PUPro1, *Lactobacillus delbrueckii* strain PUPro2, *Lacticaseibacillus casei* strain PUPro3, and *Ligilactobacillus salivarius* strain PUPro4. *L. delbrueckii* strain PUPro2 grew extremely fast. All isolates exhibited exceptional resistance to increasing concentrations of NaCl and bile salts with value of *p* >0.5. *L. delbrueckii* strain PUPro2 adhered to chicken ileum epithelial cells and demonstrated the highest viable counts of 320 colony forming units (CFUs). Antagonistic action was found in all isolates against *P. aeruginosa*, *B. subtilis*, *B. proteus*, and *S. aureus*, with value of *p* >0.5. Antibiotic susceptibility testing showed sensitivity to all the antibiotics used. Cholesterol assimilation was detected in all bacteria, with values ranging from 216.12 to 192.2 mg/dL. All isolates exhibited γ-hemolysis. In future, these bacteria might be tested for their impact on broilers meat quality and growth and can be recommended for their use as supplements for broilers diet with positive impact on poultry production.

## 1 Introduction

In Pakistan, poultry is a dynamic livestock sector which has contributed to employment opportunities to 1.5 million people in last few years due to its massive development, i.e., at the rate of 7.5%. It contributes 40% to the total annual meat production, 5.76% to agricultural sector, and 12.6% to overall GDP of Pakistan (Akbar et al., 2023). At present, Pakistan poultry turnover is 1,190 billion rupees (Alam et al., 2023). In global poultry industry, current filet yield of 16 and 22% has been recorded by the slow and fast growing chickens, respectively (Baéza et al., 2022). Poultry goods production in Pakistan during last 3 years is described in detail in Supplementary Table S1 (Henchion et al., 2021).

However, this industry has been on the brink of collapse in Pakistan as in seven tehsils of Rawalpindi and District Mansehra due to limitations associated with broilers farming (Saman et al., 2023). These limitations include high cost of broilers feed, mortality due to diseases, improper nutrition, lack of disease control programs, poor hatching conditions, improper sanitation, and poor immunity in chickens. Additionally, farmers use antibiotics to reduce enteric illnesses and increase feed conversion ratio (FCR) and body weight gain (BWG) to meet increased demand for poultry products (Desiere et al., 2018; Ogbuewu et al., 2022). As a consequence, broiler meat is saturated with carcinogenic hormones and antibiotic growth promoters (AGPs). As antibiotic abuse has virtually outlawed them, so breeders are under pressure to find more ecologically benign ways to promote the broilers growth (Arsène et al., 2021). Poor waste management practices at poultry farms expose broilers during rearing procedures to deep litter producing a filthy environment where young chicks are more likely to die immediately after delivery (Yan et al., 2021).

To compensate these losses, chicken gut microbiome is being targeted by the veterinarians and microbiologists. Replacing the gut bacteria with potential probiotics has been reported to be an emerging alternative to AGPs (Dowarah et al., 2018; Reuben et al., 2019; Melese et al., 2023). Probiotics are the live microorganisms which improve host health when given in sufficient amounts (Reid et al., 2019). Probiotics, employed in poultry industry, are the inhabitants of chicken gut in most of the cases. They improve serum calcium (Ca) and phosphorus (P) availability thus strengthening the tibial bone (Rizzoli and Biver, 2020). They boost amino acid levels in broiler chickens, improving their flavor (Yadav et al., 2018). Probiotic-fed broilers have been observed to exhibit the reduced midsection fat and increased breast muscle (Zhang et al., 2020). Metabolites of probiotics have also been found to be associated with health promotion activities such as tryptamine, short chain fatty acids (SCFAs), tryptophan, vitamins, and bacteriocins play role in immune system homeostasis, cell to cell communication, and microbiota cross talk, provide resistance to pathogens gut colonization, promote integrity, structure, and function of gut, and activate toll-like receptors (TLRs) on binding with enterocytes leading to cytokine expression (Agus et al., 2018; Khan et al., 2020; Liu et al., 2022; He et al., 2023; Li et al., 2023). In addition to this, metabolites such as lauric acid, pantothenate acid, L-glutamic acid, and N-acetyl-L-aspartic acid have been reported to positively regulate the lipid metabolism in broilers (Wu et al., 2021; Zhang et al., 2021). Probiotic supplements, at an approximate amount of 0.5 g/kg, are reported to improve meat texture and pH in broilers (Mohammed et al., 2021).

The probiotics reported so far in literature include *L. monocytogenes*, *E. faecalis*, *S. enteritidis*, *S. typhimurium*, *Bifidobacterium*, *Roseburia*, *Akkermansia*, *Propionibacterium*, *Faecalibacterium*, *L. reuteri* I2, *Pediococcus acidilactici* I5, *P. acidilactici* C3, *P. acidilactici* I8, *P. acidilactici* I13, *L. acidophilus*, *L. plantarum*, *L. brevis*, *L. salivarius* and *L. fermentum* and *Enterococcus faecium* C14, *Brevibacillus laterosporus* S62, *B. coagulans*, *B. licheniformis*, and *E. coli* (Sanders et al., 2019; Sorescu et al., 2021; Tang et al., 2021; Gupta et al., 2023; Li et al., 2023). Among these, *B. subtilis* supplementation reduced broiler belly fat in a project (Liu et al., 2020). *Lactobacillus* sp. boosted chicken feed protein and crude protein retention (Wu et al., 2019). *E. faecium* NCIMB 11181 supplementation increased FCR (Wu et al., 2019; Yang et al., 2023). *Limosilactobacillus fermentum* PC-76 and PC-10 were found to be anti-*Salmonella gallinarum* (Mehmood et al., 2023).

Under natural circumstances, young chickens are well protected through feeding on parent feces which contain efficient microflora for protection against pathogenic microbes, but the chickens being reared under commercial settings are usually deprived of such beneficial microbes due to cleanliness maintained at the hatching place, i.e., incubators. Shell contamination of pathogens and the gastric secretion of HCl at eighteenth day of incubation exacerbate the composition of gut microflora. Therefore, the post-birth probiotic feed supplementation is crucial in chickens (Lutful, 2009; Ivanova and Denisenko, 2019). Although literature reports various chicken gut-associated bacteria with different probiotic potentials, however, in majority of the cases, a single bacterium does not exhibit wide range of probiotic characteristics simultaneously and cannot be recommended as a sole nutritional tool. They can only be used in combination with other bacteria. Present study has been initiated to isolate and characterize the GIT inhabiting bacteria which might exhibit maximum potential of exerting probiotic effects on chicken health as a single bacterium. For this purpose, we selected the 10 broilers with improved performance and hypothesized that we might find wholesome probiotic bacteria from their guts. These bacteria might be administered via broiler feed as a sole zootechnical tool for the wholesomeness of meat, better FCR, high-quality egg production, healthy weight gain, effective establishment of intestinal microflora, immune system modulation, and pathogen resistance.

## 2 Materials and methods

### 2.1 Sample collection and preparation

Healthy broiler chickens (*n* = 10) at 21 days of age were purchased from a butcher and slaughtered. The chickens were washed twice with distilled water followed by 70% ethanol, before the GIT sampling. Followed this, gut was aseptically removed and washed with 0.9% NaCl. Washed gut was minced in 225 mL of ringer solution and mixed well for an hour (Musikasang et al., 2009; Shamsudin et al., 2019).

### 2.2 Isolation and culturing of probiotics

De Man–Rogosa–Sharpe agar (MRS) (Merck KGaA, 64271 Darmstadt, EMD Millipore Corporation, Germany) and Mueller Hinton agar (MHA) (M0203, FLINN SCIENTIFIC) were used for isolation and characterization of probiotics, respectively. MRS was prepared and poured into petri plates and allowed to get solidified (Kumar and Kumar, 2015; Soemarie et al., 2022; Gupta et al., 2023). The original sample (200 μL) and its six serial dilutions (10^−6^ to 10^−1^) were poured on solidified agar plates. Plates were incubated at 37°C. Bacterial colonies obtained after 24 h were enumerated to determine CFUs/ml using the formula:


$$\begin{array}{l} \mathrm{CFUs}/\mathrm{ml}\;\mathrm{of}\;\mathrm{ringers}\;\mathrm{soln}.=\\ \left(\mathrm{No}.\;\mathrm{of}\;\mathrm{colonies}\times \mathrm{dilution}\;\mathrm{factor}\right)/\mathrm{volume}\;\mathrm{of}\;\mathrm{culture} \end{array}$$

The isolated bacteria were preserved in the form of glycerol stocks for later use (Fadanka et al., 2022).

### 2.3 Molecular characterization by 16S rRNA gene sequence analysis

Using an organic extraction method, DNA was isolated from bacterial pellet (Gupta, 2019). Agarose (RXP 10121, RxBiosciences, Rockville) gels assessed DNA quality. Forty-five min after activation at 90 volts, the Gel Documentation System (4,191,354, BIO-RAD) revealed DNA bands under UV light (Green and Sambrook, 2019). Using particular primers F1 and R1 (Supplementary Table S2), 16S rDNA was amplified by polymerase chain reaction (PCR) equipment (MyGeneTM Series Thermal Cycler) (Beckers et al., 2016). The 12.5 μL master mix, 1 μL forward primer, 1 μL reverse primer, 2 μL template, 8 μL injection water, and 0.5 μL Taq polymerase were added to 20 μL PCR reaction mixture. PCR conditions used are as follows: initial denaturation at 94°C for 10–15 min, followed by 38 cycles of denaturation at 94°C for 30–40 s, annealing at 94°C for 30–40 s, extension at 72°C for 30 s, and final extension at 72°C for 10 min. The results of the PCR amplification were verified by gel electrophoresis. After inspecting the gel in UV light, the Gel Documentation System captured an image of it (Lorenz, 2012). PCR products were shipped to Macrogen, Inc., Korea, for DNA sequence analysis using Sanger chain termination method (Young, 2018).

### 2.4 Bioinformatics analysis

Sequencing results were downloaded in FASTA format. Basic Local Alignment Search Tool (BLAST) program^1^ from the National Center for Biotechnology Information (NCBI)^2^ was used to align and scan these sequences against bacterial database. Clustal Omega^3^ multiple sequence alignment was performed using NCBI consensus sequences of isolates and related bacteria. Phylogenetic tree was constructed using MEGA 11,^4^ after aligning the gaps. The neighbor joining statistical approach, maximum likelihood substitution method, and 1,000-repeat Bootstrap analysis were used to build phylogenetic trees (Sievers and Higgins, 2014).

### 2.5 Submission of DNA sequences to GenBank

Sequences of four isolates have been deposited in the publically accessible NCBI GenBank database. DNA sequences and their corresponding accession codes are listed in Supplementary Table S3.

#### 2.5.1 Growth analysis

Bacterial growth was monitored by measuring the optical density of the cultures at 600 nm (OD^600^) at different time intervals (0, 3, 6, 9, 24, 27, 30, 51, 54, 57 h) using a UV–visible spectrophotometer (BioTek Equipment, Inc.). A growth curve was obtained by plotting the OD^600^ versus time. Growth assay was performed in triplicates using synchronized cultures.

### 2.6 Biochemical characterization by RapID™ NF plus panel system

For this biochemical study, we used the Remel RapID™ NF Plus Panel kit (Thermo Scientific™) qualitative micromethod (Maloney et al., 2014). RapID™ NF Plus Panel kit screened the isolates for the presence of arginine dihydrolase (ADH), aliphatic thiol (TRD), triglyceride (EST), p-nitrophenyl-phosphoesterase (PHS), p-nitrophenyl-N-acetyl-β-D-glucosaminidase (NAG), p-nitrophenyl-D-α glucosidase (α-Glu), p-nitrophenyl-β-D-glucosidase (β-Glu), p-nitrophenyl-β-D-galactosidase (ONPG), urea hydrolysis test (URE), glucose fermentation test (GLU), proline β-naphthylamidase test (PRO), pyrrolidonyl β-naphthylamidase test (PYR), γ-glutamyl β-naphthylamidase test (GGT), tryptophan β-naphthylamidase (TRY), N-benzyl-arginine naphthylamidase test (BANA), tryptophan fermentation test (IND), and nitrate utilization test (NO_3_).

### 2.7 Assays of probiotics

All the assays performed to assess the probiotic potential of present study bacteria were carried out in triplicates. This helped to validate the observed results. Synchronized cultures were used as inoculum in triplicate experiments.

#### 2.7.1 NaCl tolerance assay

To test salt tolerance of present study bacteria, NaCl (ONLINESCIENCEMALL, Clay Palmerdale Road, Pinson, United States) concentrations of 0.2, 2, and 5% were selected. To get synchronized cultures, fresh overnight grown cultures (100 μL) were inoculated to MRS broth supplemented with different NaCl concentration. The OD^600^ was measured at 0 h. Following this, bacteria were cultured in shaking incubator (Bio-Techne, China) at 150 rpm and 37°C until the log phase was achieved. After that, bacterial growth was assessed by measuring OD^600^ at the end of exponential phase (Rocha-Ramírez et al., 2021).

#### 2.7.2 HCl tolerance assay

Present study bacterial isolates were grown for overnight at 37°C under shaking conditions. Cultures were centrifuged using centrifuge machine (HERMLE, Z380) at 6,000 rpm, 4°C, and 10 min to get the pellet. MRS medium of three different pH values, i.e., 2, 3, and 5, was prepared in three separate tubes, each tube containing 2 mL medium. The pH was adjusted with HCl (AQUABOND Inc.). Each of these tubes were inoculated with pelleted bacterial cells and incubated for 3 h at shaker at 37°C. The OD^600^ was measured at 0 h (before incubation) and 3 h (after incubation) to measure the tolerance of bacteria against different pH values (Nawaz et al., 2017).

#### 2.7.3 Bile salt tolerance assay

Bile salt tolerance was tested at 0.3% concentration of bile salt (Research Products International, United States). MRS broth without bile salts was used as control. Experimental solution was made by adding 0.3 g bile salts to 100 mL MRS broth. Both MRS broth and control were inoculated with 100 μL fresh overnight cultures. It was then cultured at 37°C and 250 rpm until the log phase was achieved. Using a spectrophotometer, OD^600^ was measured at 0 h and at log phase to test the bile salt tolerance in bacteria (Xu et al., 2020).

#### 2.7.4 Cell adhesion assay

The chicken gut was kept at 4°C for 30 min in phosphate buffer saline (PBS) (G-Biosciences, United States) to release surface mucus and was cut into four 1 cm pieces of disinfected ileum. Each component was added in probiotic cell suspension at 37°C for different time intervals, i.e., 0, 30, 60, and 90 min. Ileum was collected and macerated to remove non-adherent bacteria, seeded on MHA agar plates, and incubated for 24 h at 37°C. CFUs in each plate were counted (Reuben et al., 2019).

#### 2.7.5 Growth in the presence of pathogens

Isolates were tested against different pathogens, i.e., *Pseudomonas aeruginosa*, *Bacillus subtilis*, *Bacillus proteus*, and *Staphylococcus aureus*. The pathogen glycerol stocks (60 μL) and overnight grown cultures of probiotics (60 μL) were used as inoculum and added to MRS broth (5 mL). Control comprised of MRS broth inoculated with overnight grown probiotic culture (60 μL). Cultures were incubated under shaking conditions for 24 h at 37°C and 150 rpm. Growth of probiotics in the presence of pathogens was assessed by measuring OD^600^ (Cizeikiene and Jagelaviciute, 2021).

#### 2.7.6 Antibiotic sensitivity profiling

The disk diffusion method was used to determine the antibiotic sensitivity profile of all isolates by measuring the inhibitory zone for each antibiotic. Tested antibiotics included amoxicillin (DMS Chemical Pharmaceutical Inc., Ltd, China), azithromycin (Jenpharm Life Sciences, Pakistan), cefadroxil (Wellona Pharma, India), velosef (GlaxoSmithKline, London), kanamycin (Kanto Chemical Co., Inc., Tokyo, Japan), and augmentin (MEDSAFE, New Zealand) (Jomehzadeh et al., 2020). Three concentrations of each of these antibiotics were consulted, i.e., 5, 10, and 15 μL.

#### 2.7.7 Hemolytic assay

Autoclaved blood agar (Millipore, India) was poured into petri plates. After solidification, overnight bacterial cultures were streaked. These plates were incubated at 37°C for 24 h, and hemolytic activities were estimated. The presence of a clear, colorless, or light-yellow zone surrounding the colonies will show total red blood cell (RBC) lysis, i.e., β-hemolysis. A greenish to brownish discoloration of the media will reflect reduction of hemoglobin, i.e., α-hemolysis, and no change in media color will reflect the ϒ-hemolysis (Kumari VB et al., 2023).

#### 2.7.8 Cholesterol assay

Cholesterol breakdown characteristics were evaluated by CHOD-PAP method using cholesterol liquicolor kit (PATHOZYME DIAGNOSTICS, India). The probiotic culture cuvette received 10 μL of cholesterol standard (STD). The standard was mixed with culture and incubated at 20–25°C for 5 min. Blank cuvette contained 10 μL STD. OD was measured at 500 nm. Cholesterol concentration was measured as follows:


$$C^{STD}=553\times \Delta A\;\left(\mathrm{mg}/\mathrm{dl}\right)\;\mathrm{or},C=14.3\times \Delta A\;\left(\mathrm{mmol}/l\right)$$

$$\mathrm{Cholesterol}\;\mathrm{concentration}\;\left(\mathrm{mg}/\mathrm{dl}\right)=200\times \Delta A_{\mathrm{sample}}/\Delta A_{STD}$$

$$\mathrm{Cholesterol}\;\mathrm{concentration}\;\left(\mathrm{mmol}/l\right)=5.17\times \Delta A_{\mathrm{sample}}/\Delta A_{STD}$$

### 2.8 Statistical analysis

After each test, the findings were summarized as a mean ± SD as triplicates of each experiment were conducted separately. One-way ANOVA statistical tests were run using SPSS version 18.0. Threshold for statistical significance was set at *p* ≤ 0.05.

## 3 Results

### 3.1 Isolation of probiotics

Probiotics were isolated from chicken intestines using MRS broth. Clear bacterial colonies appeared on the plates after 24 h of incubation at 37°C.

### 3.2 Molecular characterization

Present study bacteria were identified using molecular analysis (Supplementary Figure S1). Sequences of 16S rRNA gene have been deposited in GenBank database, and accession numbers were assigned (Supplementary Table S3).

### 3.3 Phylogeny analysis

**Figure 1:**
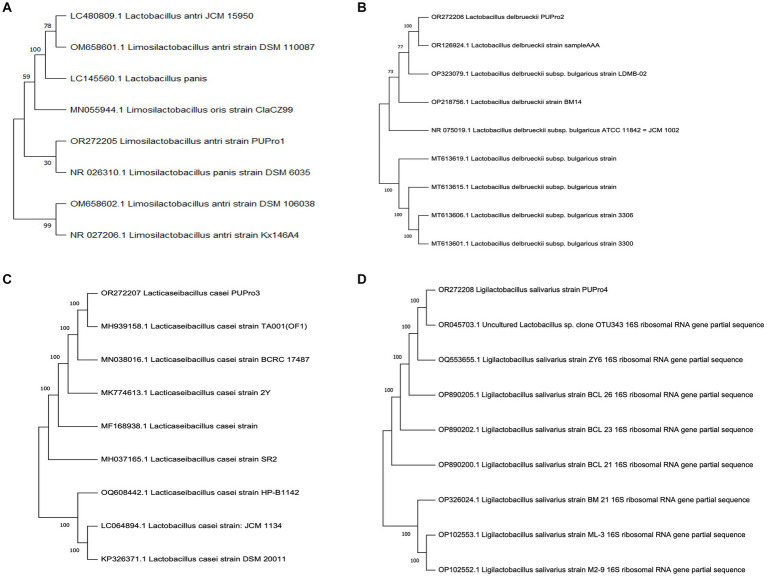
Phylogenetic trees constructed for present study bacteria using MEGA11. **(A)** *Limosilactobacillus antri* strain PUPro1. **(B)** *Lactobacillus delbrueckii* strain PUPro2. **(C)** *Lacticaseibacillus casei* strain PUPro3. **(D)** *Ligilactobacillus salivarius* strain PUPro4.

Phylogenetic analysis revealed the close relation of *L. delbrueckii* strain PUPro2 with *L. delbrueckii* strain sample AAA with bootstrap value of 100. *L. antri* strain PUPro1 and *L. panis* strain DSM 6035 both originated from the shared clade of *L. antri* strain DSM 106038 and *L. antri* strain Kx146A4. *L. casei* strain PUPro3 originated from the same branch point as that of *L. casei* strain TA001 with strong bootstrap value of 100 showing their close relatedness. *L. salivarius* strain PUPro4 was closely related to *Lactobacillus* species OTU343 through strong bootstrap value of 100 (Figure 1).

### 3.4 Growth analysis

**Figure 2:**
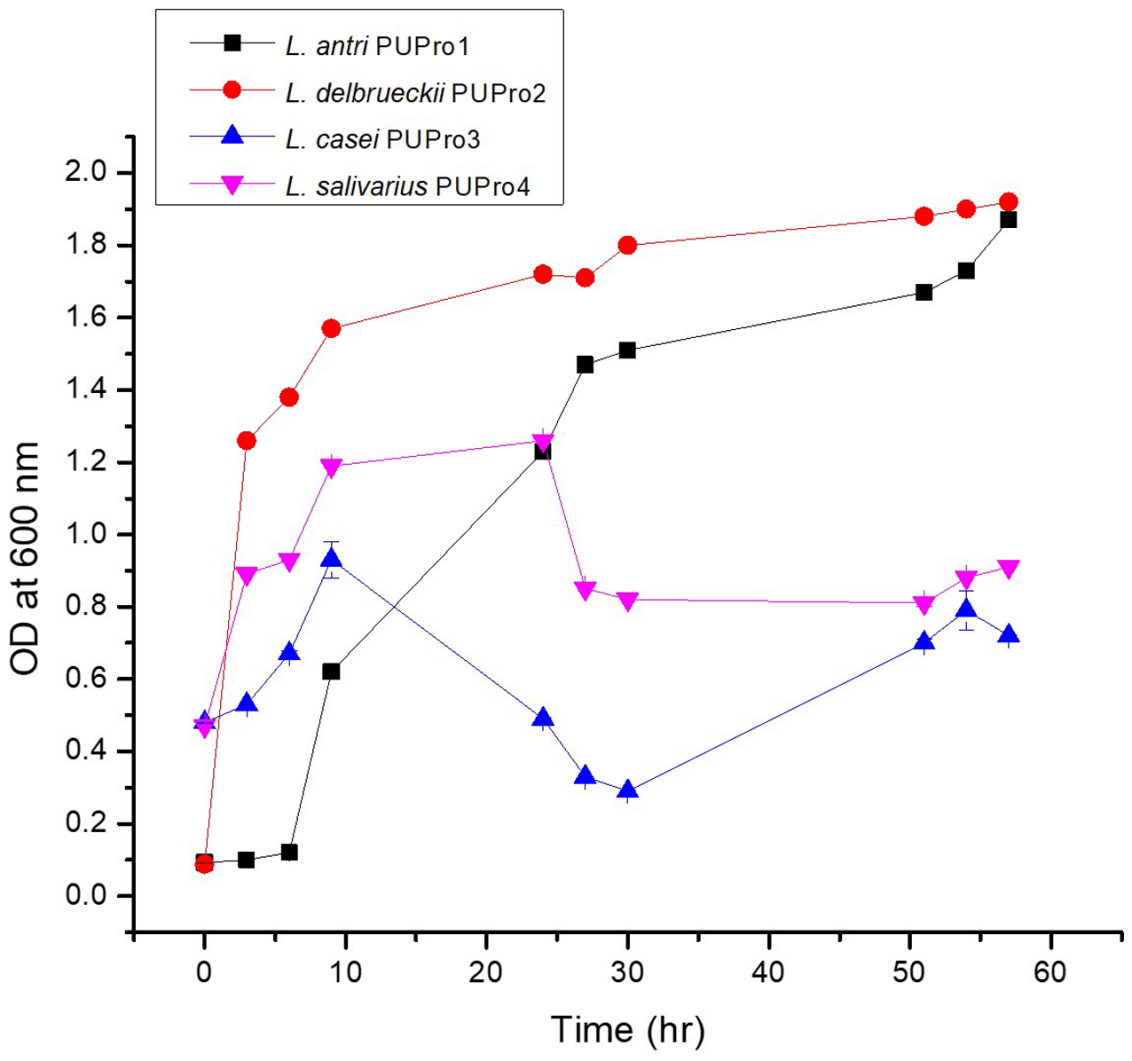
Growth curve analysis of present study bacterial isolates performed measuring OD^600^.

All bacterial isolates were fast growing. *L. antri* strain PUPro1 entered the logarithmic phase at 24 h and exited after 57 h. *L. delbrueckii* strain PUPro2 grew logarithmically from 3 to 24 h. *L. salivarius* strain PUPro4 exhibited log phase of 9–24 h. *L. casei* strain PUPro3 grew logarithmically between 6 and 24 h (Figure 2; Supplementary Tables S4, S5).

### 3.5 Biochemical characterization

All isolates were tested positive for TRD and EST tests. Only *L. antri* strain PUPro1 was positive for PHS. All isolates fermented glucose except *L. delbrueckii* strain PUPro2 and *L. casei* strain PUPro3. All isolates were nitrate-reducing and NO^3−^positive. All the isolates showed negative results for ADH, NAG, α-GLU, β-GLU, PRO, PYR, GGT, TRY, BANA, IND, and ONPG assays (Supplementary Figure S2; Supplementary Table S6).

### 3.6 Assays of probiotics

#### 3.6.1 NaCl tolerance assay

**Figure 3:**
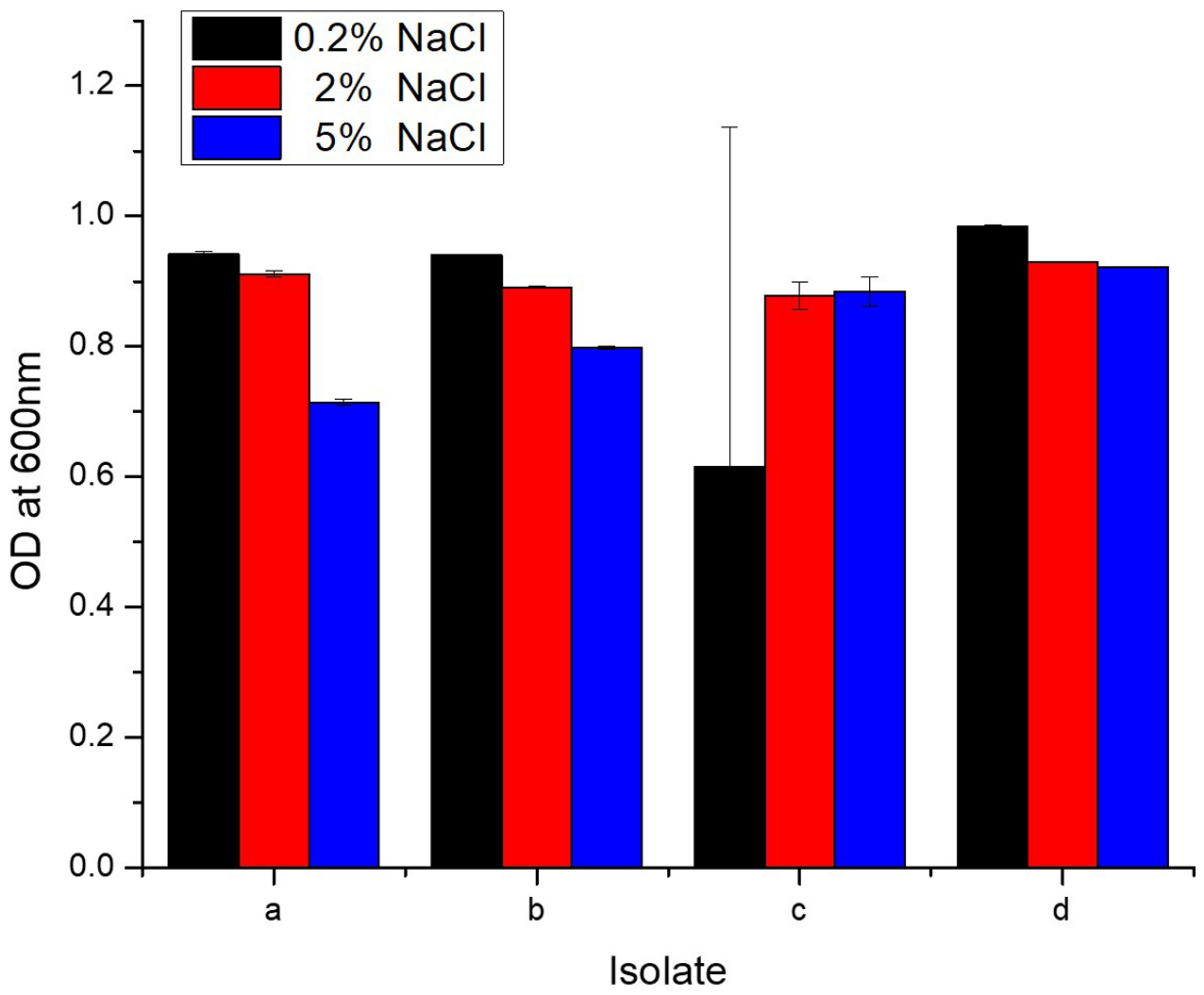
NaCl resistance potential of present study bacteria performed measuring OD^600^ at different NaCl concentrations. (a) *L. antri* strain PUPro1, (b) *L. delbrueckii* strain PUPro2, (c) *L. casei* strain PUPro3, (d) *L. salivarius* strain PUPro4.

Each isolate survived at 0.2, 2, and 5% concentration of NaCl, and OD^600^ dropped slightly as NaCl increased from 0.2 to 2 and 5% (Figure 3). *L. salivarius* strain PUPro4 had the highest tolerance at 0.2 and 2% NaCl with OD^600^ = 0.984 and 0.929, respectively, while *L. casei* strain PUPro3 had the lowest OD^600^*. L. salivarius* strain PUPro4, with an OD^600^ of 0.922, was the most tolerant to 5% NaCl. Least tolerance was observed in case of *L. antri* strain PUPro1 with 0.714 (Table 1). Statistics showed value of *p* >0.05.

**Table 1:** Survival of present study bacterial strains at 0.2, 2, and 5% NaCl concentration estimated through measuring OD^600^ in the presence of bile salts.

Isolate	OD (600 nm) at different of concentration NaCl after 24 h																	
	0.2% NaCl						2% NaCl						5% NaCl					
	1st	2nd	3rd	Mean	SD	*p*-value	1st	2nd	3rd	Mean	SD	*p*-value	1st	2nd	3rd	Mean	SD	*p*-value
*L. antri* strain PUPro1	0.947	0.939	0.941	0.942	±0.004	0.437	0.916	0.904	0.913	0.911	±0.005	0.749	0.711	0.723	0.708	0.714	±0.005	0.999
*L. delbrueckii* strain PUPro2	0.942	0.943	0.941	0.940	±0.001		0.888	0.893	0.889	0.891	±0.002		0.7999	0.794	0.801	0.798	±0.002	
*L. casei* strain PUPro3	0.013	0.919	0.913	0.615	±0.521		0.894	0.895	0.845	0.878	±0.022		0.883	0.889	0.880	0.884	±0.022	
*L. salivarius* strain PUPro4	0.980	0.987	0.985	0.984	±0.003		0.930	0.928	0.929	0.929	±0.0004		0.918	0.923	0.925	0.922	±0.0004	

#### 3.6.2 HCl tolerance assay

**Figure 4:**
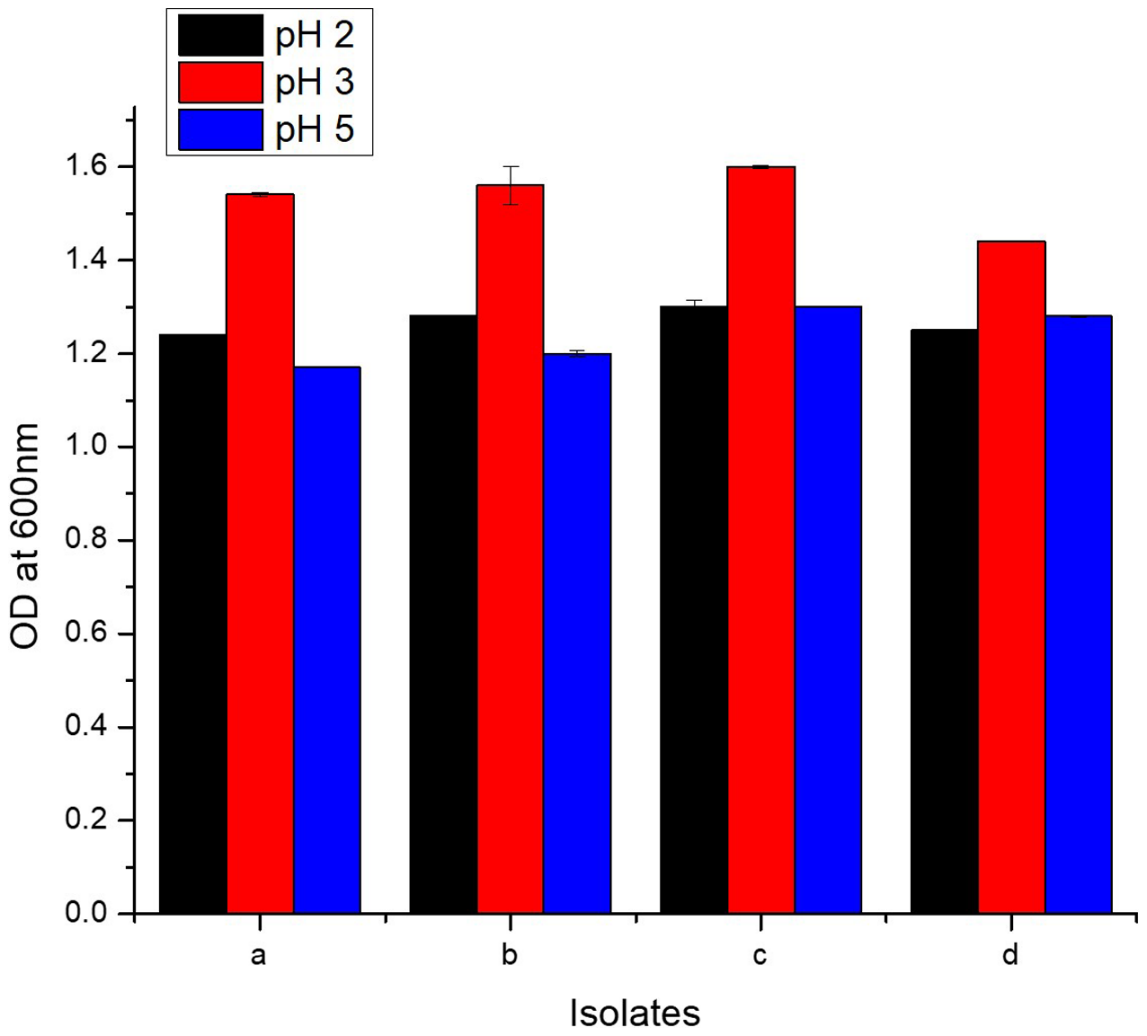
Comparison of HCl resistance potential of present study bacteria performed via measuring OD^600^ at different pH values. (a) *Limosilactobacillus antri* strain PUPro1. (b) *Lactobacillus delbrueckii* strain PUPro2. (c) *Lacticaseibacillus casei* strain PUPro3. (d)*Ligilactobacillus salivarius* strain PUPro4.

Present study bacteria were analyzed for resistance against HCl via incubation in media of three different pH values, i.e., 2, 3, and 5. All the four isolates showed maximum tolerance to HCl at pH value of 3. Highest growth measured in terms of O.D^600^ at pH 3 was 1.60 ± 0.003, 1.56 ± 0.04, 1.54 ± 0.004, and 1.44 ± 0.001 for *L. casei* strain PUPro3, *L. delbrueckii* strain PUPro2, *L. antri* strain PUPro1, and *L. salivarius* strain PUPro4, respectively (Figure 4; Supplementary Table S7). These results showed that these bacteria are capable of surviving the acidity of stomach before reaching the host chicken intestine.

#### 3.6.3 Bile salt tolerance assay

**Figure 5:**
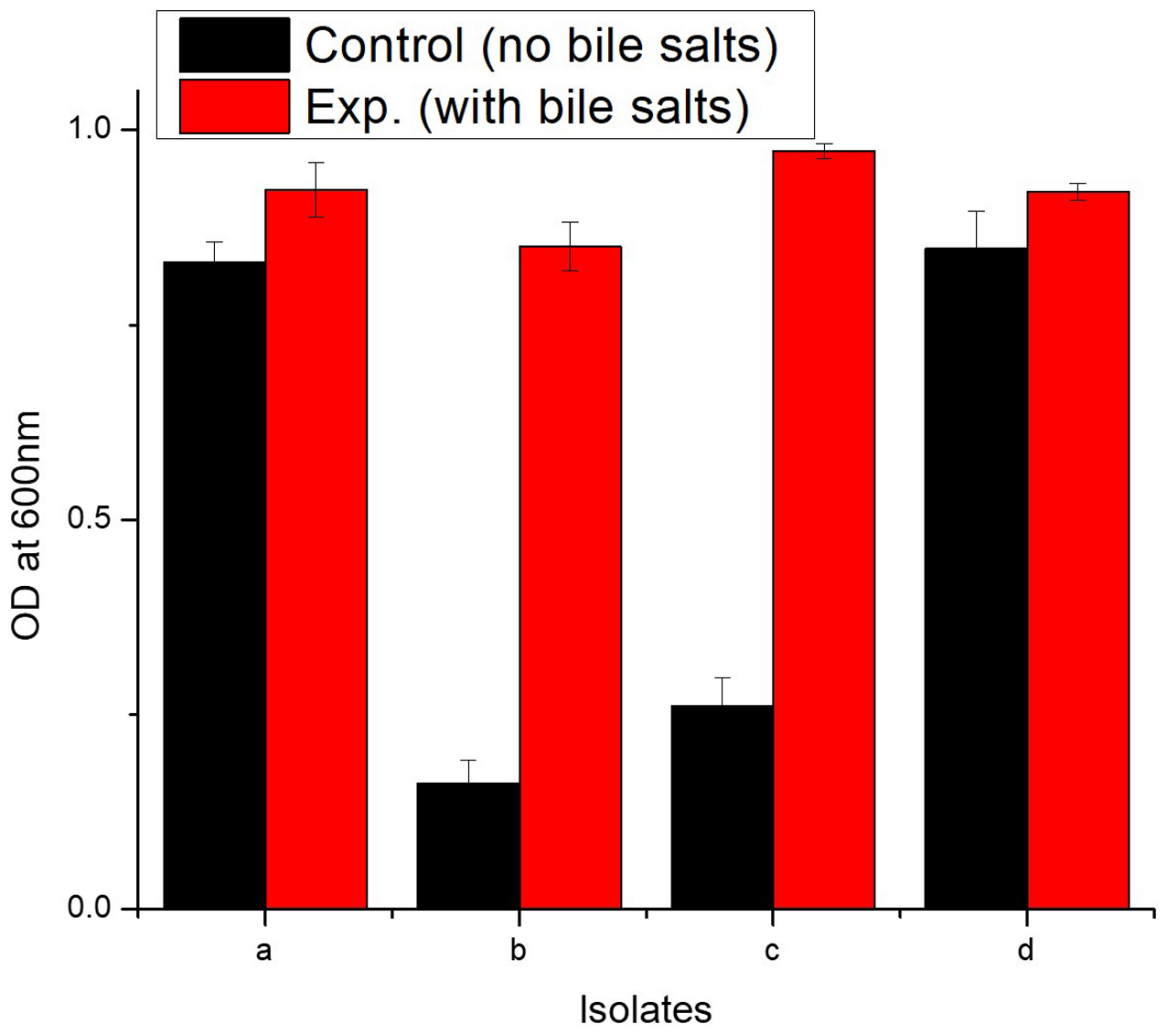
Characterization of probiotics reported in present study using bile salt tolerance assay and antimicrobial assay. (a) Comparison of OD^600^ of isolates in the absence and presence of bile salts. (b) Graphical representation of antimicrobial resistance of probiotic isolates against *Pseudomonas aeruginosa*, *Bacillus subtilis*, *Bacillus proteus*, and *Staphylococcus aureus.* (a) *L. antri* strain PUPro1, (b) *L. delbrueckii* strain PUPro2, (c) *L. casei* strain PUPro3, (d) *L. salivarius* strain PUPro4.

In bile salt tolerance test, all isolates performed well. After 24 h incubation at 37°C, the isolates with bile salts showed a marked increase in the OD^600^ value (Figure 5). *L. casei* strain PUPro3 exhibited the greatest OD^600^, i.e., 0.973 in the presence of bile salts, while *L. delbrueckii* strain PUPro2 had the lowest value of 0.851 (Table 2). Statistical analysis revealed significant results with value of p greater than 0.05.

**Table 2:** Bile salt resistance estimated in present study bacterial strains through measurement of OD^600^ in the presence and absence of bile salts.

Isolates	OD of control at log phase (600 nm)						OD with bile salts at log phase (600 nm)					
	1st	2nd	3rd	Mean	SD	*p*-value	1st	2nd	3rd	Mean	SD	*p*-value
*L. antri* strain PUPro1	0.81	0.861	0.820	0.83	± 0.027	0.996	0.92	0.961	0.89	0.923	± 0.035	0.987
*L. delbrueckii* strain PUPro2	0.192	0.132	0.160	0.161	± 0.030		0.851	0.82	0.883	0.851	± 0.031	
*L. casei* strain PUPro3	0.273	0.22	0.291	0.261	± 0.036		0.985	0.964	0.972	0.973	± 0.010	
*L. salivarius* strain PUPro4	0.871	0.881	0.792	0.848	± 0.048		0.921	0.933	0.911	0.921	± 0.011	

#### 3.6.4 Cell adhesion assay

**Figure 6:**
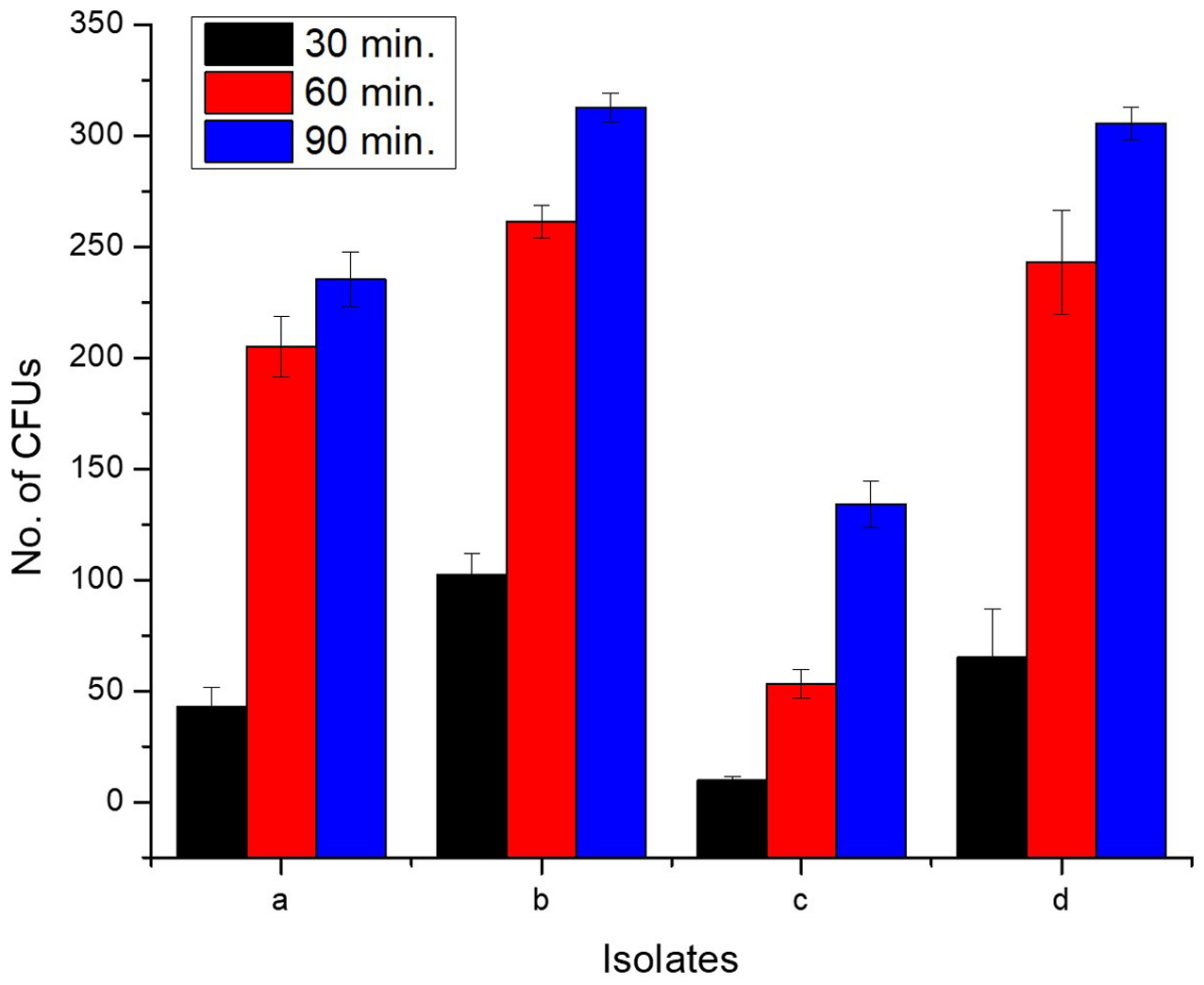
Comparison of CFUs of present study bacteria formed at different time intervals during cell adhesion assay indicating their ileum adhering tendency. (a) *Limosilactobacillus antri* strain PUPro1. (b) *Lactobacillus delbrueckii* strain PUPro2. (c) *Lacticaseibacillus casei* strain PUPro3. (d) *Ligilactobacillus salivarius* strain PUPro4.

All isolates adhered to chicken ileum epithelial cells, and the number of CFUs increased as incubation time raised from 0 to 90 min (Supplementary Figure S3; Figure 6). Incubation for 0 min yielded zero CFU*. L. delbrueckii* strain PUPro2 adhered chicken ileum epithelial cells best and had the highest CFUs, i.e., 102.33, 261.33, and 312.67 after 30, 60, and 90 min of incubation, respectively. Least tendency of adherence was observed in case of *L. casei* strain PUPro3 (Table 3). The results were significant with *p* > 0.05.

**Table 3:** Analysis of intestinal adhesion potential of present study isolates through determination of colony forming units (CFUs) at 0, 30, 60, and 90 min in cell adhesion assay.

Isolates	No. of CFUs at 0 min incubation						No. of CFUs at 30 min incubation						No. of CFU at 60 min Incubation						No of CFU at 90 min Incubation					
	1st	2nd	3rd	Mean	SD	*p*-value	1st	2nd	3rd	Mean	SD	*p*-value	1st	2nd	3rd	Mean	SD	*p*-value	1st	2nd	3rd	Mean	SD	*p*-value
*L. antri* strain PUPro1	0	0	0	0	0	0.00	33	49	47	43	± 8.717	0.82	201	220	194	205	± 13.453	0.956	222	246	238	235.33	± 12.220	0.988
*L. delbrueckii* strain PUPro2	0	0	0	0	0		93	102	112	102.33	± 9.504		261	269	254	261.33	± 7.505		307	311	320	312.67	± 6.658	
*L. casei* strain PUPro3	0	0	0	0	0		12	8	9	9.66	± 2.08		47	53	60	53.33	± 6.506		139	122	141	134	± 10.440	
*L. salivarius* strain PUPro4	0	0	0	0	0		49	57	90	65.33	± 21.73		249	263	217	243	± 23.579		311	297	309	305.67	± 7.571	

#### 3.6.5 Growth in the presence of pathogens

**Figure 7:**
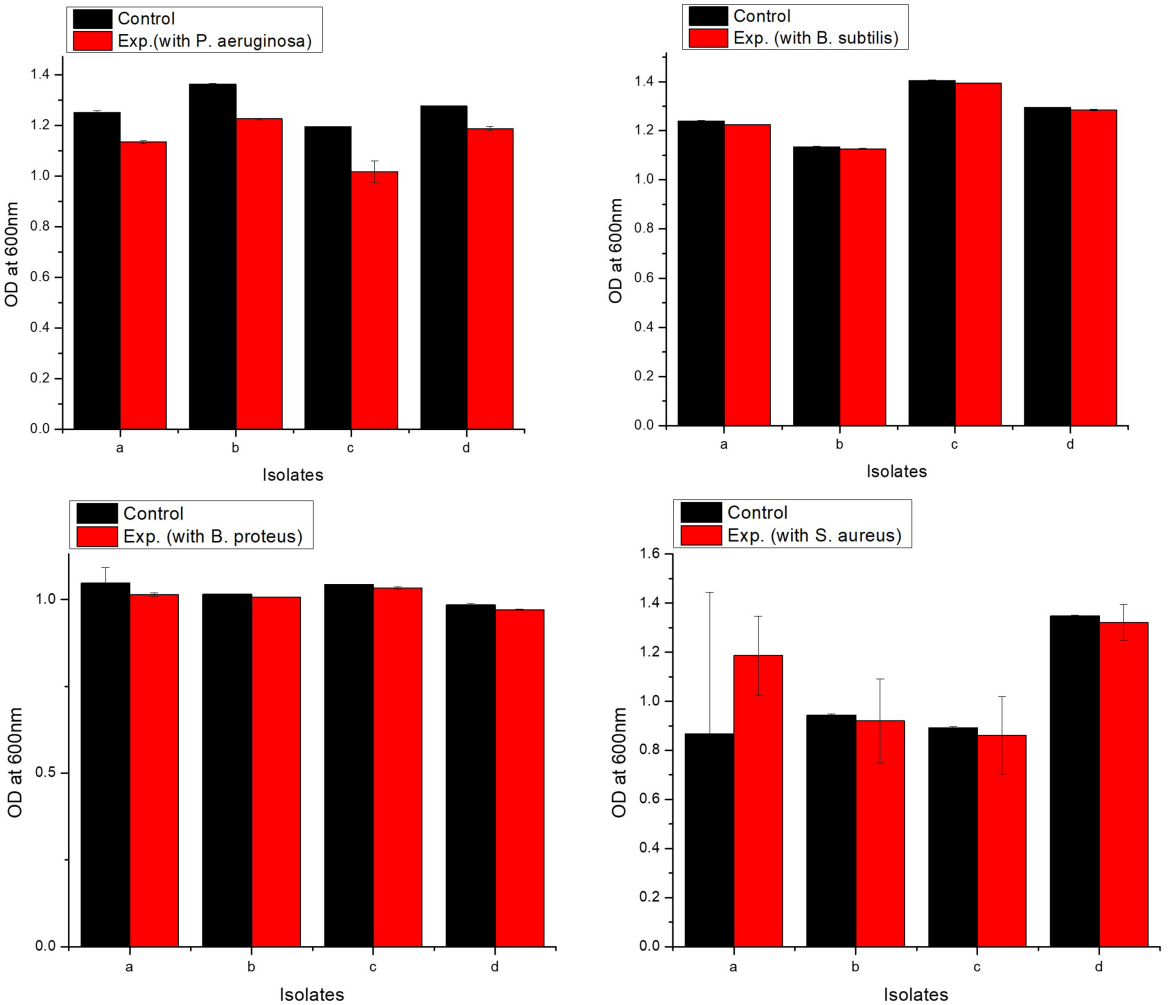
Analysis of resistance against pathogenic bacteria in present study isolates. Control comprised of medium and bacterium, while experimental tube contained medium, bacterium, and pathogen. (a) *L. antri* strain PUPro1, (b) *L. delbrueckii* strain PUPro2, (c) *L. casei* strain PUPro3, (d) *L. salivarius* strain PUPro4.

All isolates showed excellent tolerance against pathogens. *L. delbrueckii* strain PUPro2 had the highest tolerance against *Pseudomonas aeruginosa* with OD^600^ of 1.226, whereas *L. casei* strain PUPro3 had the lowest resistance with OD^600^ = 1.017 (Supplementary Table S8). *L. casei* strain PUPro3 exhibited highest resistance against *B. subtilis* with OD^600^ = 1.394, while *L. delbrueckii* strain PUPro2 showed least tolerance with OD^600^ of 1.127 (Supplementary Table S9). *L. casei* strain PUPro3 had highest OD^600^ value of 1.033 against *B. proteus* (Supplementary Table S10). *L. salivarius* strain PUPro4 had the highest OD^600^ (1.321) against *Staphylococcus aureus*, while in case of *L. casei* strain PUPro3, the value was 0.861 (Supplementary Table S11). Statistical analysis demonstrated the *p*-value of above 0.05 (Figure 7).

#### 3.6.6 Antibiotic sensitivity profiling

**Figure 8:**
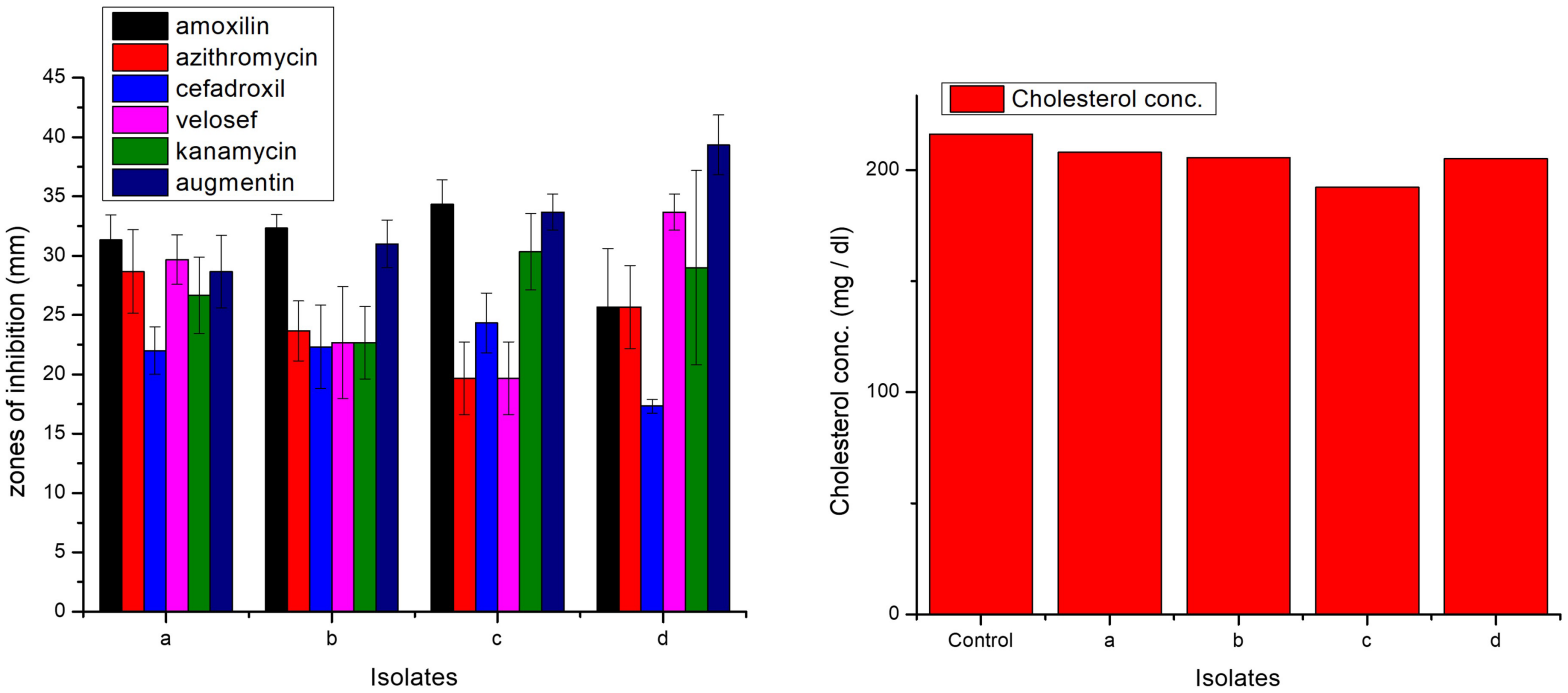
Comparison of zones of inhibition exhibited by present study bacteria against different antibiotics and comparison of cholesterol degradation potential of present study bacteria evaluated by CHOD-PAP method using cholesterol liquicolor kit. (a) *L. antri* strain PUPro1, (b) *L. delbrueckii* strain PUPro2, (c) *L. casei* strain PUPro3, (d) *L. salivarius* strain PUPro4.

All isolates were antibiotic-sensitive and exhibited zones of inhibition (Figure 8). In case of amoxicillin, cefadroxil, and kanamycin, highest zones of inhibition were observed in *L. casei* strain PUPro3, i.e., 34.33, 24.33, and 30.33 mm, respectively, while for azithromycin in *L. antri* strain PUPro1 (28.67 mm). For velosef and augmentin, largest zones were observed in *L. salivarius* strain PUPro4, i.e., 33.67 and 39.33 mm (Supplementary Table S12). Significant *p* > 0.05 were obtained for all the antibiotics.

#### 3.6.7 Hemolytic assay

Since there was no change in color of media, so all isolates were found to show ϒ-hemolysis (Supplementary Table S13). No zones were observed around the colonies (Supplementary Figure S4), indicating no RBC lysis, making these isolates the safest and best.

#### 3.6.8 Cholesterol assay

Among the four present study bacteria, *L. casei* strain PUPro3 significantly degraded cholesterol from 216.12 to 192.2 mg/dL. *L. antri* strain PUPro1 showed the lowest cholesterol assimilation, i.e., 208 mg/dL (Figure 8).

## 4 Discussion

Poultry serves as major source of animal protein in the form of meat and eggs all over the world. In 2018, per person consumption of broiler meat has been estimated to be 14 kg globally and 48 kg in USA (Thorp, 2021). In Pakistan, both the private and public sectors have increased their poultry investment from 1.28 billion $ in 2015 to 4.47 billion $ in 2018 (Aslam et al., 2020). Protein production from animal agriculture in a sustainable way is becoming crucial need because the global food demand is expected to increase by 35 to 56% between 2010 and 2050 (Van Dijk et al., 2021; Castro et al., 2023), but these days poultry industry is suffering from major challenges which can be overcome through direct-fed microbial (DFM) probiotics which might equip chickens with beneficial properties such as tolerance to heat stress, reduction in foodborne illnesses, competitive exclusion of pathogens, enhanced immunity, and prebiotics with positive physiological impact (Abd El-Hack et al., 2020; Ebeid et al., 2021).

Keeping in view the role of probiotics in overcoming the challenges of poultry industry, present project was initiated to isolate bacteria from broiler chicken GIT and their *in vitro* assessment for probiotic potential. Bacteria qualifying the *in vitro* assessment assays, as described in methodology of this study, might contribute to upgrade the economic status of Pakistan associated with poultry performance by providing hygienic and proper broilers diet in cost effective way. These might serve for the production of disease-resistant broilers with reduce cooking, pressing, and shear losses. They may also enhance bone mass, meat quality (amino acids, fat content, protein content, texture), and FCR (Pourakbari et al., 2016; Fekadie et al., 2022).

In addition to abovementioned benefits, the prebiotics derived from these probiotics also positively regulate the chicken metabolism in variety of ways, e.g., compound probiotics have been observed to contribute to growth performance of broilers exposed to heat stress, through increased rate of arginine, amino sugar, alanine, aspartate and glutamate, and beta alanine biosynthesis (Zhang et al., 2023). Metabolomics has also confirmed the formation of same metabolites in chicken intestine as formed in the presence of antibiotics, i.e., amino acids, sugars, organic acids, disaccharides, and trisaccharides, confirming their use as alternative to antibiotics (Li et al., 2023). Among the probiotics reported in the literature, *Lactobacillus* have been found to have strong involvement in the regulation of gut metabolites which in turn improves homoeostasis in chickens. In a study, chickens were fed on *L. acidophilus* supplemented diet which positively affected the amino acid and lipid metabolism along with increased production of immunoglobulin G (IgG) (Chen et al., 2023). Another study has reported enhanced concentration of acetate in broiler chickens fed with diet supplemented with *L. sali*var*ius*. When this probiotic was combined with phytobiotics, it reduced extended-spectrum beta-lactamase (ESBL) producing *E. coli* (Ren et al., 2019). In a study, broilers fed with *L. plantarum-*derived metabolites considerably increased height of villi and growth (Gao et al., 2017). Dietary supplementation with *L. plantarum* along with fructooligosaccharide (FOS) showed increased levels of short chain fatty acids (SCFAs), IgA, and IgG and efficient growth performance in *E. coli* O78 challenged broilers (Ding et al., 2019). Another study has reported increased expression of genes involved in sulfur and nitrogen metabolism, flagella assembly and chemotaxis, and vitamin and cofactor synthesis, in lincomycin exposed broilers, supplemented with *L. plantarum* P-8 (Gao et al., 2017). Literature has also reported efficient role of *L. plantarum-*derived postbiotics in enhancing carcass yield, immunity, intestinal microbial composition, and growth performance of broilers exposed to heat stress (Humam et al., 2019).

In addition to above health benefits, probiotics through interfering the metabolic pathways of host chickens enable them to withstand heat and oxidative stresses. Probiotics, in intestinal epithelial cells, activate the heat shock proteins (HSPs), glutathione S-transferase (GST), and glutathione (GSH) and inactivates iNOS, COX-2, NF-KB, and toll-like receptor-4 (TLR-4) when there is high reactive oxygen species (ROS) accumulation. ROS are produced in intestine under oxidative stress. Activation of GST by probiotics results in production of GSH which serves as an antioxidant and removes ROS. Inhibition of iNOS by probiotics inhibits synthesis of nitric oxide synthase (NOS) resulting in decreased nitric oxide (NO) thus reducing ROS. Inhibition of COX-2, NF-KB, and TLR-4 reduces heat shock-induced inflammation in intestinal epithelium thus protecting the digestive system from permanent damage. To reduce the heat-induced epithelium disruption, probiotics accelerate the production of mucous and tight junction-adherent junction (TJ-AJ) pathway which seals intracellular space between neighboring cells (Abd El-Hack et al., 2020).

Keeping in view, these beneficial effects of probiotics, we attempted to isolate bacteria from chicken GIT. Four fast growing isolates were processed for detailed *in vitro* characterization. These isolates were identified to be *L. antri*, *L. delbrueckii*, *L. casei*, and *L. sali*var*ius*, respectively. As all of these had different species and also showed variations during characterization, so were designated as different strains, i.e., PUPro1, PUPro2, PUPro3, and PUPro4, respectively. Our study is consistent with the previous one because most of the broilers have been reported to have *Lactobacilli* as gut flora (Boonkumklao et al., 2006). *L. casei, L. johnsonii, L. acidophilus, L. crispatus, L. salivarius,* and *L. aviaries* are the most reported *Lactobacillus* species in poultry (Sanders and Klaenhammer, 2001; Tharmaraj and Shah, 2003; Neville and O’Toole, 2010; Pieniz et al., 2014).

All isolates were found fast growing. Log phases comprising of 24–57 h, 3–51 h, 6–24 h, and 9–24 h were observed in *L. antri* strain PUPro1, *L. delbrueckii* strain PUPro2, *L. casei* strain PUPro3, and *L. sali*var*ius* strain PUPro4, respectively. These findings are in line with previously reported chicken gut-associated bacteria which exhibited a rapid growth (Vinderola et al., 2002; Śliżewska and Chlebicz-Wójcik, 2020).

Literature describes various biochemical features of probiotics (Bansal et al., 2019; Samedi and Charles, 2019; Abid et al., 2022), but this is the first study to use the RapID™ NF Plus Panel for biochemical testing. The ability of a bacterium to use arginine as a carbon source can be tested biochemically by ADH test (Kwon et al., 2005). In present analysis, none of the isolates tested positive for ADH. All isolates were positive in EST and GLU test results. In the EST test, lipid break down releases fatty acids which drop the pH and change the color (MacFaddin, 2000). With the exception of *L. antri* strain PUPro1, all of the probiotic strains tested positive for PHS assay (Gilardi et al., 1975). None of the isolates showed positive results for GGT, NAG, α-GLU, β-GLU, PRO, BANA, IND, TRY, PYR, ONPG, and URE tests that detects the presence of glutamyl aminopeptidase, p-nitrophenyl-N-acetyl-*β*-D-glucosaminidase, ρ-nitrophenyl-D-glucosidase, ρ-nitrophenyl-β-D-glucosidase, proline aminopeptidase, N-benzyl-arginine aminopeptidase, potential of tryptophan to indole conversion, tryptophan aminopeptidase, pyrrolidonyl aminopeptidase, ρ-nitrophenyl-β-D-galactosidase, and urea hydrolysis potential, respectively (Westley et al., 1967; Mulczyk and Szewczuk, 1970; Gilardi et al., 1975; Kilian and Bülo, 1976; Nagatsu et al., 1976; Humble et al., 1977; Holt et al., 1984; Kwon et al., 2005). *L. antri* strain PUPro1 and *L. salivarius* strain PUPro4 tested positive for NO_3_ test that identifies nitrate reductase activity (Mulczyk and Szewczuk, 1970).

All the isolates were found to be resistant to NaCl at 0.2, 2, and 5% concentration*. L. salivarius* strain PUPro4 was the most resistant, with OD^600^ of 0.984, 0.929, and 0.922 for 0.2, 2, and 5% NaCl concentration, respectively. A previous study indicated that LAB from milk and milk products, meats, chicken dung, and other sources can grow in 1–10% NaCl treatments (Hoque et al., 2010; Shehata et al., 2016). Our findings are consistent with the literature and imply that present study bacteria can survive higher concentrations of NaCl and stress in vitro (Ye et al., 2020).

Probiotic strains must bind to host intestinal cells for better colonization (Collado et al., 2007). Probiotics’ antimicrobial and cholesterol-lowering benefits require strong colon epithelial cell attachment (Shokryazdan et al., 2014). In this study, all isolates adhered to chicken ileum epithelial cells with increased viability over 90 min *L. casei* strain PUPro3, with a maximum CFUs count of 134, showed the lowest adhesion ability. Our findings are consistent with those of literature that observed rumen LAB isolates’ adherence increased with incubation period (Setyawardani et al., 2014; Jose et al., 2015).

Stay of probiotics in different parts of chicken GIT varies as per the pH values of these parts. Generally, pH varies between 2 and 6.5, i.e., pH values for crop, proventriculus, gizzard, small intestine, and large intestine have been reported to be 4.8, 4.4, 2.6, 6.2, and 6.3, respectively (Svihus, 2014). Keeping in view these values, acidity tolerance of present study bacteria was estimated at three pH values, i.e., 2, 3, and 5. Maximum survival during interval of 3 h was observed in *L. delbrueckii* strain PUPro2 (pH = 2), in *L. antri* strain PUPro1, *L. delbrueckii* strain PUPro2 and *L. casei* strain PUPro3 (pH = 3), and in *L. casei* strain PUPro3 (pH = 6). No survival was observed in *L. antri* strain PUPro1 at pH = 6. These results were according to the previous research study reporting good survival of *L. salivarius* and *L. fermentum* at pH = 2.5 for 3 h (Hutari et al., 2011). Other studies have also reported survival of probiotics at the same pH (Reuben et al., 2019; Salehizadeh et al., 2020; Gupta et al., 2023). These findings confirmed the survival of present study bacteria in chicken gut during passage through stomach.

Hydrophobic bile salts play crucial roles in digestive system including fat emulsification and resistance against antimicrobials. So, when taken orally, probiotics must survive the severe environment of small intestine (Olejnik et al., 2005). In chicken gut, bile salt concentration varies, i.e., 0.0085% (caecum), 0.17% (duodenum), and 0.7% (jejunum). Typical 0.3% bile salt level has been considered in many probiotic bile salt tolerance studies (Shokryazdan et al., 2014; García-Hernández et al., 2016). The current study’s isolates survived in 0.3% bile salts and exhibited enhanced growth after 24 h. In this study, *L. casei* strain PUPro3 had the highest OD value of 0.973 in the presence of bile salts. Present finding is in accordance with previous literature reporting tolerance to 0.3% concentration of bile salts (Shin et al., 2008; Salehizadeh et al., 2020). Our findings also support prior research that revealed bile salts increased probiotic proliferation (Reuben et al., 2019).

The chicken industry loses a lot of revenue due to zoonotic and foodborne disease illnesses (Nallala et al., 2017). Potential probiotics need to have antimicrobial activity against such pathogens. All of the present study isolates were highly antagonistic to four distinct pathogens: *Pseudomonas aeruginosa, Bacillus subtilis, Bacillus proteus*, and *Staphylococcus aureus.* Antimicrobial activity against *P. aeruginosa*, *B. subtilis*, *B. proteus*, and *S. aureus* was highest for *L. delbrueckii* strain PUPro2, *L. casei* strain PUPro3, *L. casei* strain PUPro3, and *L. salivarius* strain PUPro4. This finding is consistent with previous study. Our results are also supported by the observation that chicken-derived probiotics have a broad-spectrum antagonistic impact against a wide range of infections (García-Hernández et al., 2016; Oyewole et al., 2018).

Antibiotic-resistant genes in probiotic could cause gut infections, so antibiotic profiling of isolated bacteria was crucial (Nallala et al., 2017). All isolates were ecologically benign and showed unique inhibitory zones against augmentin, azithromycin, amoxicillin, kanamycin, cefadroxil, and velosef. The obtained inhibitory zones had diameters between 17.33 mm and 39.33 mm. The literature reports antibiotic susceptibility in chicken probiotic cultures against antibiotics such as penicillin, ampicillin, chloramphenicol, ceftriaxone, and novobiocin supporting our findings (Gueimonde Fernández et al., 2013; Anandharaj and Sivasankari, 2014; Puniya et al., 2016; Dowarah et al., 2018). However, contrary to our findings, the literature also reports resistance against kanamycin, aminoglycosides and glycopeptides, streptomycin, gentamicin, and vancomycin in *Lactobacilli* (Jose et al., 2015; Zavišić et al., 2023).

According to FAO guidelines, probiotic microbial strains should not pose any host safety risks (Borras et al., 2021). Hemolytic activity destroys the protective epithelial layer, allowing infections to penetrate easily. Non-pathogenic strains must be non-hemolytic (De Vuyst et al., 2003). None of the isolates tested in this study produced the toxic chemical hemolysin, and all were non-hemolytic. Non-hemolytic LAB strains have been reported in the literature, which is in line with our findings (Chang et al., 2013; Oloyede and Afolabi, 2013; Oyewole et al., 2018).

Hypercholesterolemia or high blood cholesterol is a primary cause of deaths caused by heart diseases in poultry which can be decreased by lowering serum cholesterol (Shehata et al., 2016). Cholesterol lowering potential of isolates was measured using a cholesterol liquicolor kit. All of the isolated bacteria showed degradation potential toward cholesterol. The *L. casei* strain PUPro3 was the most effective at degrading cholesterol, reducing cholesterol levels from 216.12 to 192.2 mg/dL. *L. antri* strain PUP1 degraded cholesterol at a rate of just 208 mg/dL. These results are consistent with the literature (Miremadi et al., 2014; Shehata et al., 2016).

## 5 Conclusion

Probiotic strains separated from their natural host are more likely to establish themselves in the GIT and exert their therapeutic benefits. Present study is significant because the bacteria isolated might have potential as antibiotic substitutes and broiler productivity boosters. To further test and confirm their possible effect on broilers meat quality, they can be cultured for the estimation of CFU production followed by their immobilization in approximate quantity on any suitable substrate. This will enhance their shelf life. Afterward, these probiotics can be fed to broilers through feed supplementation. In addition to this, probiotic metabolomics and its integration with proteomics might also prove helpful in getting insight into their possible contributions to sustainable poultry production.

## Data availability statement

The datasets presented in this study can be found in online repositories. The names of the repository/repositories and accession number(s) can be found in the article/Supplementary material.

## Author contributions

FS: Data curation, Writing – original draft. FM: Conceptualization, Writing – original draft. AS: Formal analysis, Writing – original draft. SS: Writing – original draft. SA: Formal analysis, Writing – original draft. AQ: Writing – review & editing.

## Footnotes

^1^ https://blast.ncbi.nlm.nih.gov

^2^ https://www.ncbi.nlm.gov

^3^ https://www.ebi.ac.uk

^4^ https://www.megasoftware.net
