# Supplementary material for: Isolation and in vitro assessment of chicken gut microbes for probiotic potential
Source: Front Microbiol. 2024 Jan 29;15:1278439. doi: 10.3389/fmicb.2024.1278439 (PMC10860760; doi:10.3389/fmicb.2024.1278439)
Supplement: Supplementary file 1 [file Data_Sheet_1.docx]

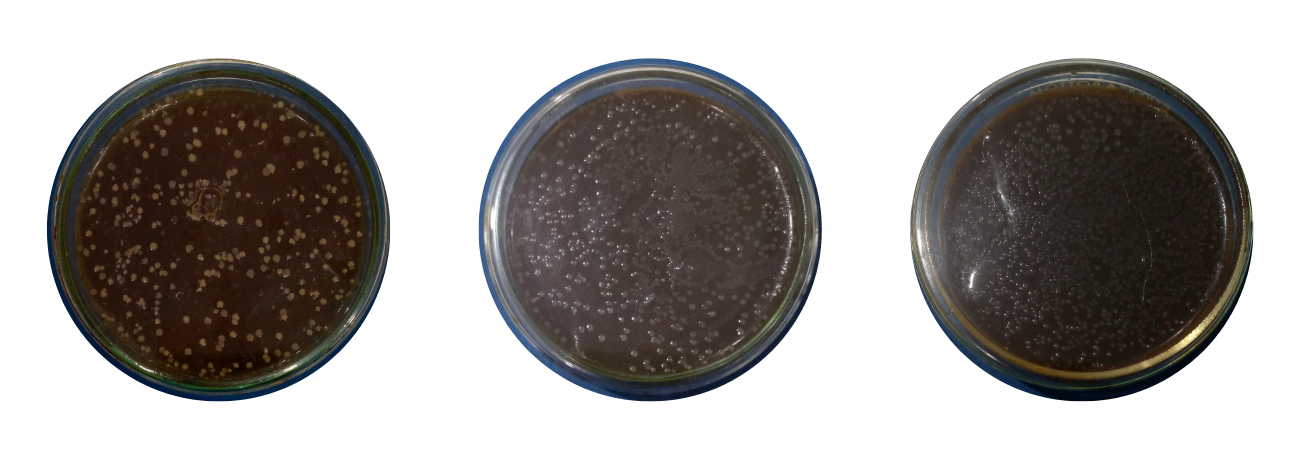


**(a)**

**
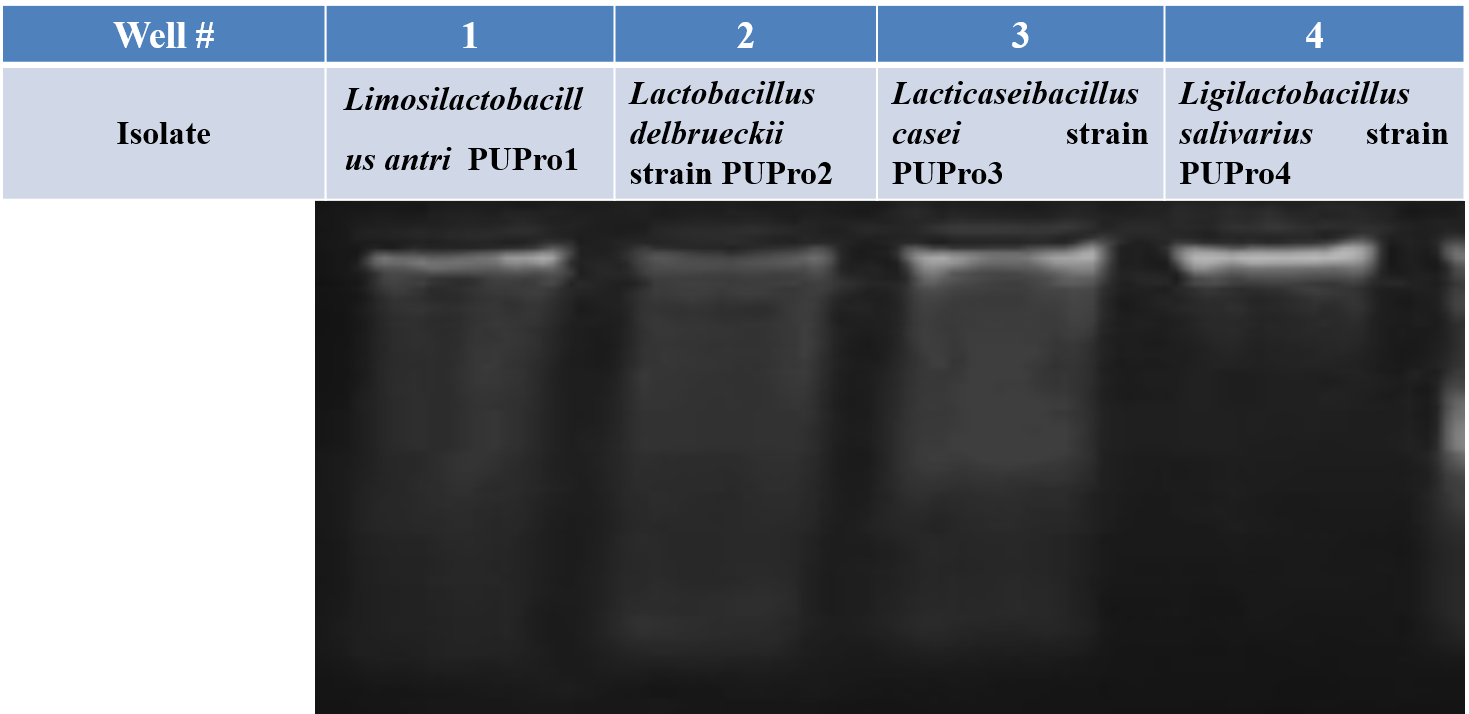
**

**(b)**

**
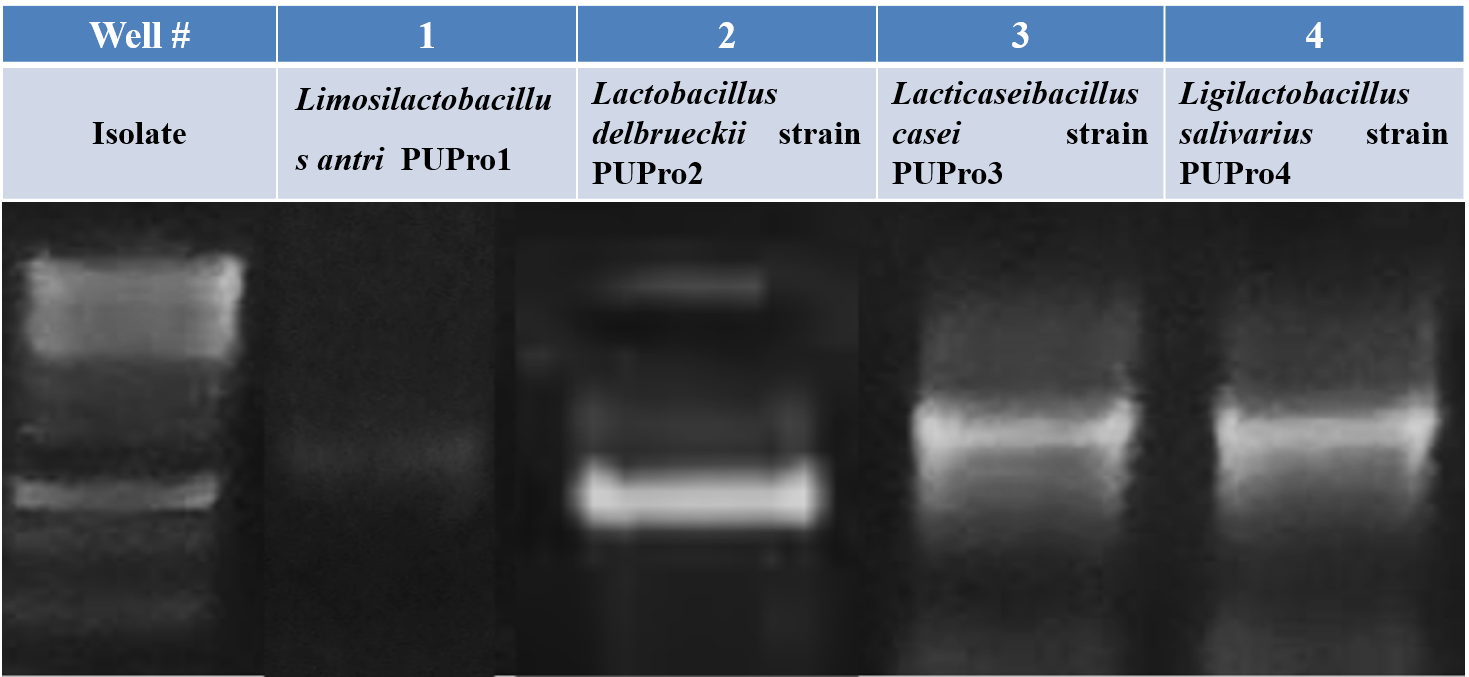
**

**(c)**

**Supplementary data Figure 1:** Results of isolation and molecular characterization of probiotics isolated form chicken gut in present study

(a) Probiotics CFUs obtained by using serial dilution technique (b) Extracted DNA of probiotics resolved on agarose gel (c) PCR amplicons of 16S rRNA gene of probiotics resolved on agarose gel

**
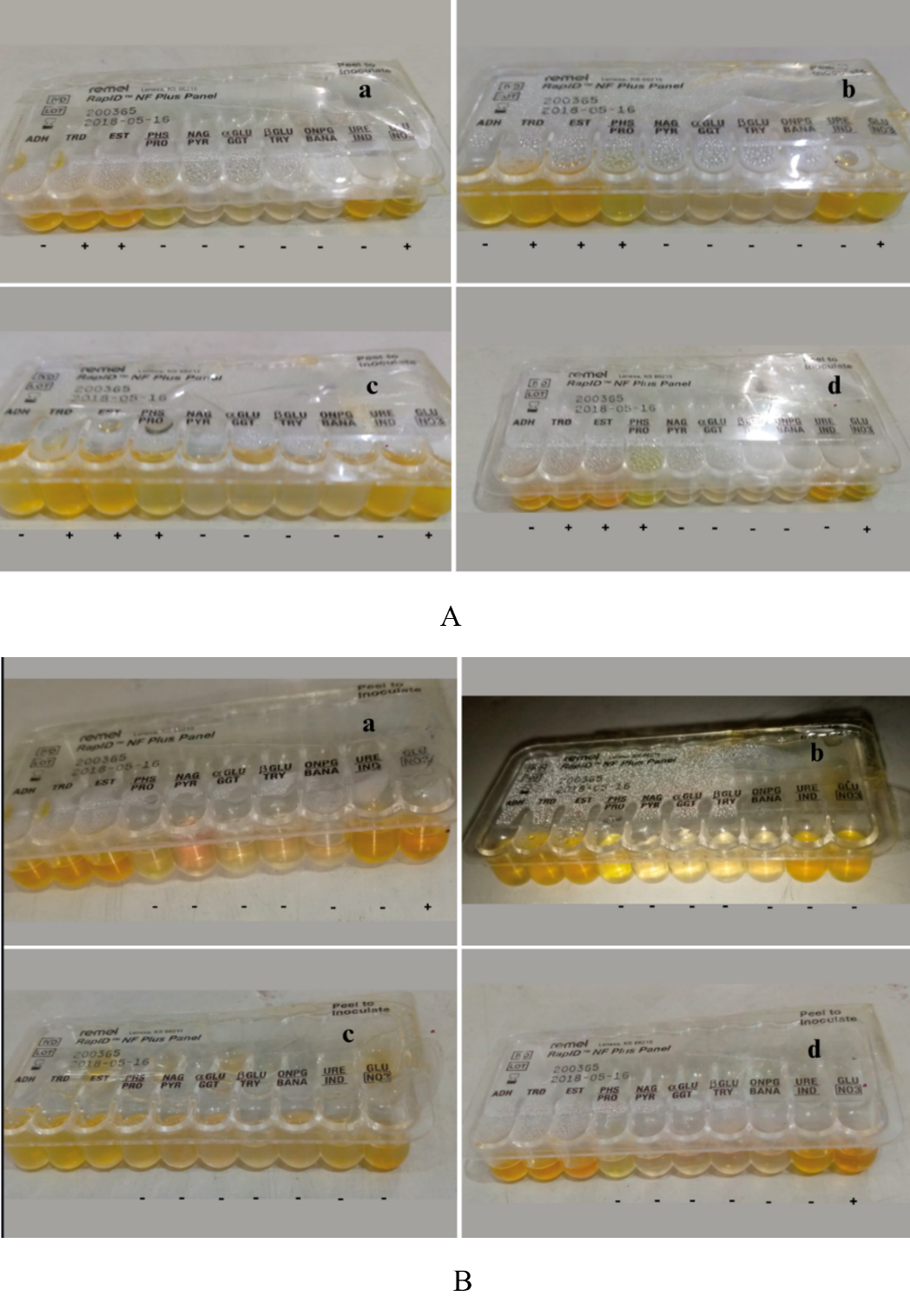
**

**Supplementary data Figure 2:** Biochemical characterization of present study bacteria using RapID ^TM^ NF Plus System

(A) RapID ^TM^ NF Plus Panel before reagent addition, (B) RapID ^TM^ NF Plus Panel after reagent addition, (a): *L. antri* strain PUPro1, b: *L. delbrueckii* strain PUPro2, c: *L. casei* strain PUPro3, d: *L. salivarius* strain PUPro4, e: *E. durans* strain PUPro5, f: *L. acidophilus* strain PUPro6, g: *E. durans* strain PUPro7, h: *L. salivarius* strain PUPro8),


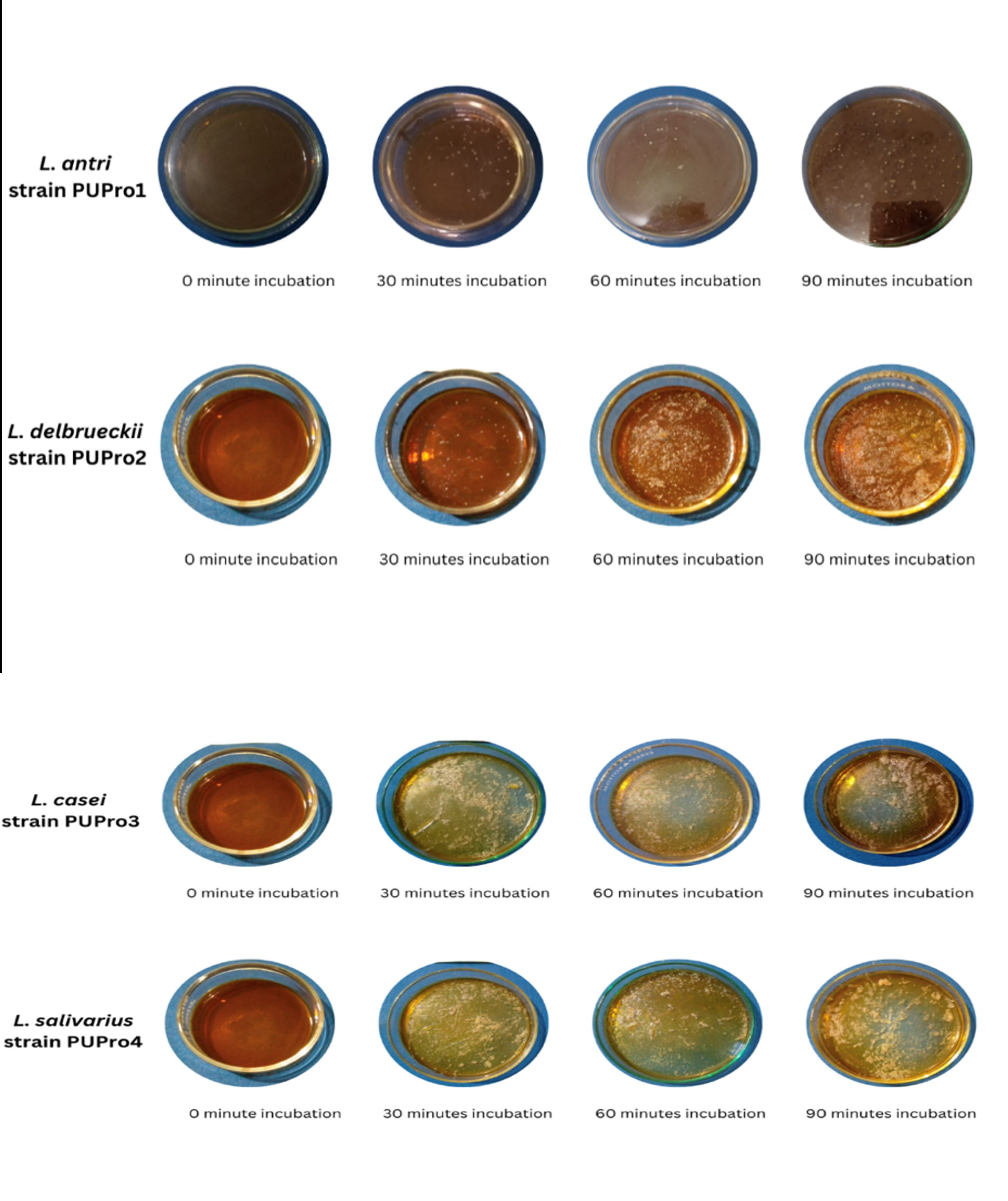


**Supplementary data Figure 3:** Characterization of present study bacteria via cell adhesion assay

**
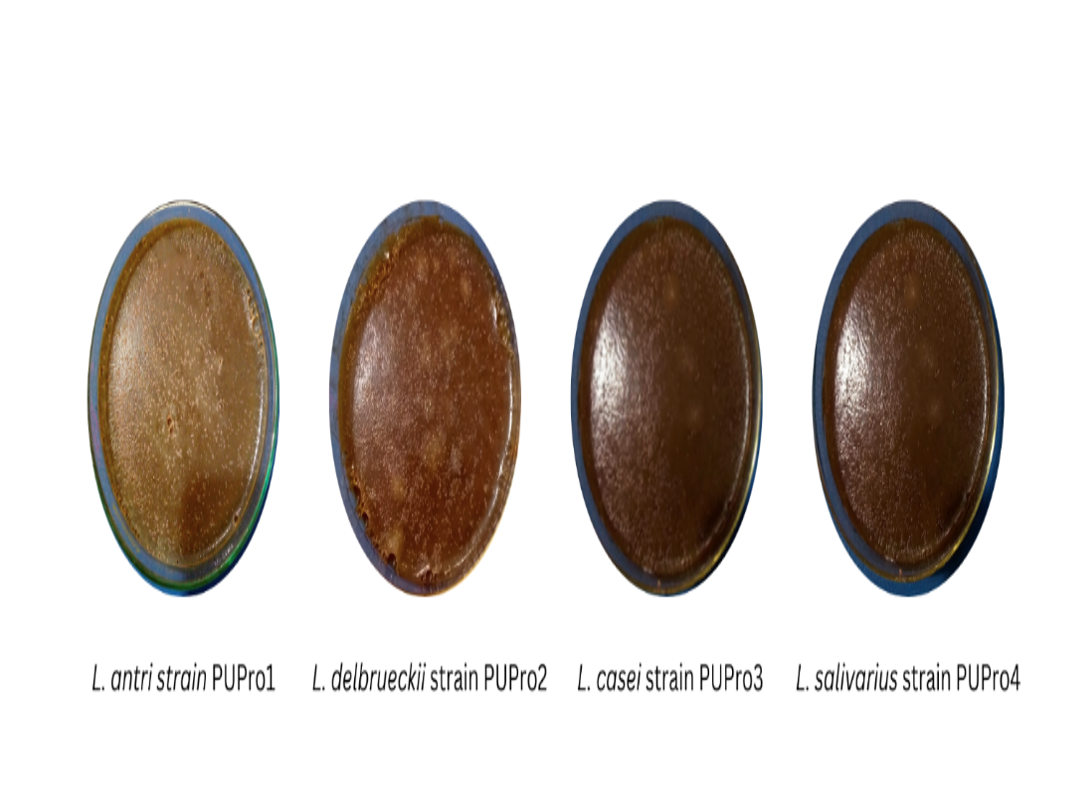
**

**Supplementary data Figure 4:** Characterization of present study bacteria by hemolysis assay.

**Supplementary data Table 1:** Poultry goods production in Pakistan during 2017-2020

| **Type** | **Unit** | **2017-2018** | **2018-2019** | **2019-2020** |
| --- | --- | --- | --- | --- |
| **Layer type** | Millions | 52.25 | 55.91 | 59.82 |
| **Broiler type** | Millions | 1,057.65 | 1,163.42 | 1,279.76 |
| **Eggs** | Millions | 13,779 | 14,719 | 15,723 |
| **Meat** | Thousand Tonnes | 1,270.69 | 1,395.02 | 1,531.60 |

**Supplementary data Table 2:** 16SrRNA gene specific primers used in present study and their Tm, GC content and product size

| **Primer name** | **5’-3’ sequence** | **Primer T_m_** | **GC content** | **PCR product size** |
| --- | --- | --- | --- | --- |
| 16s rRNA (799F) | AACMGGATTAGATACCCKG | 55.7^o^C | 47.06% | 600 bp |
| 16s rRNA (1391R) | GACGGGCGGTGWGTRCA | 64.2^o^C | 73.33% |  |

**Supplementary data Table 3:** Probiotic isolates, accession numbers assigned and 16S rRNA gene sequences

| # | **Bacterium** | **Accession No.** | **Sequence** |
| --- | --- | --- | --- |
| 1 | *Limosilactobacillus antri*  strain PUPro1 | OR272205 | GGTGGCAAGCGTTATCCGGATTTATTGGGCGTAAAGCGAGCGCAGGCGGTTGCTTAGGTCTGATGTGAAAGCCTTCGGCTTAACCGAAGAAGTGCATCGGAAACCGGGCGACTTGAGTGCAGAAGAGGACAGTGGAACTCCATGTGTAGCGGTGGAATGCGTAGATATATGGAAGAACACCAGTGGCGAAGGCGGCTGTCTGGTCTGCAACTGACGCTGAGGCTCGAAAGCATGGGTAGCGAACAGGATTAGATACCCTGGTAGTCCATGCCGTWAACGATGAGTGCTAGGTGTTGGAGGGTTTCCGCCCTTCAGTGCCGAAGCTAACGCATTAAGCACTCCGCCTGGGGAGTACGACCGCAAGGTTGAAACTCAAAGGAATTGACGGGGGCCCGCACAAGCGGTGGAGCATGTGGTTTAATTCGAAGCTACGCGAAGAACCTTACCAGGTCTTGACATCTTGCGCCAACCTCAGAGATGAGGCGTTCCCTTCGGGGACGCAAAGACAGGTGGTGCATGGTCGTCGTCAGCTCGTGTCGTGAGATGTTGGGTTAAGTCCCGCAACGAGCGCAACCCTTGTTACTAGTTGCCAGCATTCAGTTGGGCACTCTAGTGAGACTGCCGGTGACAAACCGGAGGAAGGTGGGGACGACGTCAGATCATCATGCCCCTTATGACCTGGGCTACACACGTGCTACAATGGCCGGTACAACGAGCAGCTAACCCGCGAGGGTGTGCAAATCTCTTAAAGCCGGTCTCAGTTCGGACTGCAGTCTGCAACTCGACTGCACGAAGTCGGAATCGCTAGTAATCGCGGATCAGCATGCCGCGGTGAATACGTTCCCGGGCCTTGTACACACCGCCCGTCACACCATGGAAKTTTGCAATGCCCAAAGTCAGTGGCCTAACCATTATGGAGGGASCTGCCTAAGGCAGGGCAGATGACTGGGGTGAAGTCGT |
| 2 | *Lactobacillus delbrueckii* strain PUPro2 | OR272206 | GATTTATTGGGCGTAAAGCGAGCGCAGGCGGAATGATAAGTCTGATGTGAAAGCCCACGGCTCAACCGTGGAACTGCATCGGAAACTGTCATTCTTGAGTGCAGAAGAGGAGAGTGGAATTCCATGTGTAGCGGTGTAATGCGTAGATATATGGAAGAACACCAGTGGCGAAGGCGGCTCTCTGGTCTGCAACTGACGCTGAGGCTCGAAAGCATGGGTAGCGAACAGGATTAGATACCCTGGTAGTCCATGCCGTAAACGATGAGCGCTAGGTGTTGGGGACTTTCCGGTCCTCAGTGCCGCAGCAAACGCATTAAGCGCTCCGCCTGGGGAGTACGACCGCAAGGTTGAAACTCAAAGGAATTGACGGGGGCCCGCACAAGCGGTGGAGCATGTGGTTTAATTCGAAGCAACGCGAAGAACCTTACCAGGTCTTGACATCCTGTGCTACACCTAGAGATAGGTGGTTCCCTTCGGGGACGCAGAGACAGGTGGTGCATGGCTGTCGTCAGCTCGTGTCGTGAGATGTTGGGTTAAGTCCCGCAACGAGCGCAACCCTTGTCTTTAGTTGCCATCATTAAGTTGGGCACTCTAAAGAGACTGCCGGTGACAAACCGGAGGAAGGTGGGGATGACGTCAAGTCATCATGCCCCTTATGACCTGGGCTACACACGTGCTACAATGGGCAGTACAACGAGAAGCGAACCCGCGAGGGTAAGCGGATCTCTTAAAGCTGTTCTCAGT |
| 3 | *Lacticaseibacillus casei*  strain PUPro3 | OR272207 | GGTTTTTTAAGTCTGATGTGAAAGCCCTCGGCTTAACCGAGGAAGCGCATCGGAAACTGGGAAACTTGAGTGCAGAAGAGGACAGTGGAACTCCATGTGTAGCGGTGAAATGCGTAGATATATGGAAGAACACCAGTGGCGAAGGCGGCTGTCTGGTCTGTAACTGACGCTGAGGCTCGAAAGCATGGGTAGCGAACAGGATTAGATACCCTGGTAGTCCATGCCGTAAACGATGAATGCTAGGTGTTGGAGGGTTTCCGCCCTTCAGTGCCGCAGCTAACGCATTAAGCATTCCGCCTGGGGAGTACGACCGCAAGGTTGAAACTCAAAGGAATTGACGGGGGCCCGCACAAGCGGTGGAGCATGTGGTTTAATTCGAAGCAACGCGAAGAACCTTACCAGGTCTTGACATCTTTTGATCACCTGAGAGATCAGGTTTCCCCTTCGGGGGCAAAATGACAGGTGGTGCATGGTTGTCGTCAGCTCGTGTCGTGAGATGTTGGGTTAAGTCCCGCAACGAGCGCAACCCTTATGACTAGTTGCCAGCATTGAGTTGGGCACTCTAGTAAGACTGCCGGTGACAAACCGGAGGAAGGTGGGGATGACGTCAAATCATCATGCCCCTTATGACCTGGGCTACACACGTGCTACAATGGATGGTACAACGAGTTGCGAGACCGCGAGGTCAAGCTAATCTCTTAAAGCCATTCTCAGTTCGGACTGTAGGCTGCAACTCGCCTACACGAAGTCGGAATCGCTAGTAATCGCGGATCAGCACGCCG |
| 4 | *Ligilactobacillus salivarius*  strain PUPro4 | OR272208 | GCAAGTCACGGCTAACTACGTGCCAGCAGCCGCGGTAATACGTAGGTGGCAAGCGTTGTCCGGATTTATTGGGCGTAAAGGGAACGCAGGCGGTCTTTTAAGTCTGATGTGAAAGCCTTCGGCTTAACCGGAGTAGTGCATTGGAAACTGGAAGACTTGAGTGCAGAAGAGGAGAGTGGAACTCCATGTGTAGCGGTGAAATGCGTAGATATATGGAAGAACACCAGTGGCGAAAGCGGCTCTCTGGTCTGTAACTGACGCTGAGGTTCGAAAGCGTGGGTAGCAAACAGGATTAGATACCCTGGTAGTCCACGCCGTAAACGATGAATGCTAGGTGTTGGAGGGTTTCCGCCCTTCAGTGCCGCAGCTAACGCAATAAGCATTCCGCCTGGGGAGTACGACCGCAAGGTTGAAACTCAAAGGAATTGACGGGGGCCCGCACAAGCGGTGGAGCATGTGGTTTAATTCGAAGCAACGCGAAGAACCTTACCAGGTCTTGACATCCTTTGACCACCTAAGAGATTAGGCTTTCCCTTCGGGGACAAAGTGACAGGTGGTGCATGGCTGTCGTCAGCTCGTGTCGTGAGATGTTGGGTTAAGTCCCGCAACGAG  CGCAACCCTTGTTGTCAGTTGCCAGCATTAAGTTGGGCACTCTGGCGAGACTGCCGGTGACAAACCGGAGGAAGGTGGGGACGACGTCAAGTCATCATGCCCCTTATGACCTGGGCTACACACGTGCTACAATGGACGGTACAACGAGTCGCAAGACCGCGAGGTTTAGCTAATCTCTTAAAGCCGTTCTCAGTTCGGATTGTAGGCTGCAACTCGCCTACATGAAGTCGGAATCGCTAGTAATCGCGAATCAGCATGTCGC |

**Supplementary data Table 4:** Growth measured in terms of OD^600^ at different time intervals in present study bacteria

| Isolate | Time (hr.) | | | | | | | | | |
| --- | --- | --- | --- | --- | --- | --- | --- | --- | --- | --- |
|  | **0** | **3** | **6** | **9** | **24** | **27** | **30** | **51** | **54** | **57** |
| *L. antri* strain PUPro1 | 0.091 | 0.103 | 0.125 | 0.626 | 1.235 | 1.480 | 1.520 | 1.690 | 1.760 | 1.869 |
|  | 0.093 | 0.101 | 0.126 | 0.619 | 1.233 | 1.470 | 1.530 | 1.670 | 1.730 | 1.880 |
|  | 0.092 | 0.104 | 0.123 | 0.622 | 1.231 | 1.470 | 1.500 | 1.650 | 1.720 | 1.864 |
| *L. delbrueckii* strain PUPro2 | 0.089 | 1.241 | 1.384 | 1.573 | 1.728 | 1.724 | 1.804 | 1.900 | 1.912 | 1.930 |
|  | 0.087 | 1.229 | 1.382 | 1.575 | 1.731 | 1.719 | 1.802 | 1.890 | 1.911 | 1.929 |
|  | 0.088 | 1.240 | 1.381 | 1.573 | 1.730 | 1.714 | 1.810 | 1.870 | 1.901 | 1.926 |
| *L. casei* strain PUPro3 | 0.485 | 0.539 | 0.671 | 0.892 | 0.495 | 0.331 | 0.298 | 0.700 | 0.828 | 0.727 |
|  | 0.486 | 0.536 | 0.671 | 0.991 | 0.489 | 0.329 | 0.293 | 0.690 | 0.826 | 0.729 |
|  | 0.489 | 0.542 | 0.688 | 0.912 | 0.491 | 0.330 | 0.291 | 0.710 | 0.729 | 0.733 |
| *L. salivarius* strain PUPro4 | 0.479 | 0.891 | 0.939 | 1.196 | 1.268 | 0.862 | 0.828 | 0.800 | 0.886 | 0.912 |
|  | 0.477 | 0.889 | 0.937 | 1.194 | 1.269 | 0.855 | 0.827 | 0.820 | 0.882 | 0.911 |
|  | 0.478 | 0.890 | 0.938 | 1.192 | 1.268 | 0.862 | 0.829 | 0.810 | 0.883 | 0.909 |

**Supplementary data Table 5:** Mean growth of present study isolates estimated on the basis of OD^600^ measurement at different time intervals

| Time  (hr.) | Isolates | | | | | | | | |
| --- | --- | --- | --- | --- | --- | --- | --- | --- | --- |
|  | ***L. antri* strain PUPro1** | | ***L. delbrueckii* strain PUPro2** | | ***L. casei* strain PUPro3** | | ***L. salivarius* strain PUPro4** | | **p-value** |
|  | **Mean** | **Standard Deviation** | **Mean** | **Standard Deviation** | **Mean** | **Standard Deviation** | **Mean** | **Standard Deviation** |  |
| 0 | 0.092 | ±0.001 | 0.088 | ±0.001 | 0.48 | ±0.002 | 0.47 | ±0.001 | < 0.05 |
| 3 | 0.10 | ±0.001 | 1.26 | ±0.006 | 0.53 | ±0.003 | 0.89 | ±0.001 |  |
| 6 | 0.12 | ±0.001 | 1.38 | ±0.001 | 0.67 | ±0.009 | 0.93 | ±0.001 |  |
| 9 | 0.62 | ±0.003 | 1.57 | ±0.0009 | 0.93 | ±0.05 | 1.19 | ±0.002 |  |
| 24 | 1.23 | ±0.002 | 1.72 | ±0.001 | 0.49 | ±0.003 | 1.26 | ±0.0005 |  |
| 27 | 1.47 | ±0.005 | 1.71 | ±0.005 | 0.33 | ±0.001 | 0.85 | ±0.004 |  |
| 30 | 1.51 | ±0.015 | 1.80 | ±0.004 | 0.29 | ±0.003 | 0.82 | ±0.001 |  |
| 51 | 1.67 | ±0.02 | 1.88 | ±0.015 | 0.7 | ±0.01 | 0.81 | ±0.01 |  |
| 54 | 1.73 | ±0.02 | 1.9 | ±0.006 | 0.79 | ±0.056 | 0.88 | ±0.002 |  |
| 57 | 1.87 | ±0.008 | 1.92 | ±0.002 | 0.72 | ±0.003 | 0.91 | ±0.001 |  |

**Supplementary data Table 6:** Biochemical characterization of present study bacteria using RapID ^TM^ NF Plus Panel system

| **Isolates** | **Biochemical Characterization** | | | | | | | | | | | | | | | | | |
| --- | --- | --- | --- | --- | --- | --- | --- | --- | --- | --- | --- | --- | --- | --- | --- | --- | --- | --- |
|  | **Before Reagent Addition** | | | | | | | | | | | **After Reagent Addition** | | | | | | |
|  | **ADH** | **TRD** | **EST** | **PHS** | **NAG** | **α-GLU** | **β-GLU** | **ONPG** | **URE** | **GLU** | **PRO** | | **PYR** | **GGT** | **TRY** | **BANA** | **IND** | **NO3** |
| ***L. antri* strain PUPro1** |  |  |  |  |  |  |  |  |  |  |  | |  |  |  |  |  |  |
| ***L. delbrueckii* strain PUPro2** |  |  |  |  |  |  |  |  |  |  |  | |  |  |  |  |  |  |
| ***L. casei* strain PUPro3** |  |  |  |  |  |  |  |  |  |  |  | |  |  |  |  |  |  |
| ***L. salivarius* strain PUPro4** |  |  |  |  |  |  |  |  |  |  |  | |  |  |  |  |  |  |

Red = negative result, Yellow = positive result

**Supplementary data Table 7:** HCl resistance estimated in present study bacterial isolates through measuring OD^600^ at 0 hr. and 3 hr. of incubation in media of pH values 2, 3 and 5

| **Isolates** | **pH** | **Initial conc. (O.D^600^ at 0 hr.)** | **p-value** | **Final conc. (OD^600^ at 3 hr.)** | **p-value** |
| --- | --- | --- | --- | --- | --- |
|  |  |  |  |  |  |
| ***L. antri* strain PUPro1** | **2** | 0.12 ± 0.001 | 0.05 | 1.24 ± 0.0002 | 0.05 |
|  | **3** | 0.18 ± 0.002 |  | 1.54 ± 0.004 |  |
|  | **5** | 0.17 ± 0.002 |  | 1.17 ± 0.001 |  |
| ***L. delbrueckii* strain PUPro2** | **2** | 0.10 ± 0.001 |  | 1.28 ± 0.0013 |  |
|  | **3** | 0.15 ± 0.0015 |  | 1.56 ± 0.04 |  |
|  | **5** | 0.14 ± 0.003 |  | 1.20 ± 0.006 |  |
| ***L. casei* strain PUPro3** | **2** | 0.13 ± 0.004 |  | 1.30 ± 0.014 |  |
|  | **3** | 0.19 ± 0.001 |  | 1.60 ± 0.003 |  |
|  | **5** | 0.20 ± 0.015 |  | 1.30 ± 0.001 |  |
| ***L. salivarius* strain PUPro4** | **2** | 0.12 ± 0.001 |  | 1.25 ± 0.003 |  |
|  | **3** | 0.17 ± 0.003 |  | 1.44 ± 0.001 |  |
|  | **5** | 0.16 ± 0.015 |  | 1.28 ± 0.002 |  |

**Supplementary data Table 8:** Resistance of present study isolates against *Pseudomonas aeruginosa* estimated through antimicrobial resistance assay

| Isolates | *Pseudomonas aeruginosa* | | | | | | | | | | | |
| --- | --- | --- | --- | --- | --- | --- | --- | --- | --- | --- | --- | --- |
|  | **OD OF CONTROL AT (600nm)** | | | | | | **OD OF ISOLATES & PATHOGEN AT (600nm)** | | | | | |
|  | **I** | **II** | **III** | **Mean** | **Standard Deviation** | **p-value** | **I** | **II** | **III** | **Mean** | **Standard Deviation** | **p-**  **value** |
| *L. antri* strain PUPro1 | 1.249 | 1.236 | 1.251 | 1.25 | ± 0.008 | < 0.05 | 1.139 | 1.129 | 1.134 | 1.134 | ± 0.005 | < 0.05 |
| *L. delbrueckii* strain PUPro2 | 1.361 | 1.368 | 1.362 | 1.363 | ±0. 003 |  | 1.224 | 1.22 | 1.226 | 1.226 | ± 0.003 |  |
| *L. casei* strain PUPro3 | 1.197 | 1.193 | 1.195 | 1.195 | ± 0.002 |  | 1.011 | 1.09 | 1.017 | 1.017 | ± 0.043 |  |
| *L. salivarius* strain PUPro4 | 1.286 | 1.281 | 1.284 | 1.277 | ± 0.002 |  | 1.191 | 1.176 | 1.188 | 1.188 | ± 0.007 |  |

| Isolates | *Bacillus subtilis* | | | | | | | | | | | |
| --- | --- | --- | --- | --- | --- | --- | --- | --- | --- | --- | --- | --- |
|  | **OD OF CONTROL AT (600nm)** | | | | | | **OD OF ISOLATES & PATHOGEN AT (600nm)** | | | | | |
|  | **I** | **II** | **III** | **Mean** | **Standard Deviation** | **p-**  **value** | **I** | **II** | **III** | **Mean** | **Standard Deviation** | **p-**  **value** |
| *L. antri* strain PUPro1 | 1.237 | 1.24 | 1.244 | 1.24 | ± 0.003 | < 0.05 | 1.223 | 1.224 | 1.226 | 1.224 | ± 0.001 | < 0.05 |
| *L. delbrueckii* strain PUPro2 | 1.137 | 1.136 | 1.133 | 1.135 | ± 0.002 |  | 1.129 | 1.128 | 1.124 | 1.127 | ± 0.002 |  |
| *L. casei* strain PUPro3 | 1.403 | 1.409 | 1.402 | 1.404 | ± 0.003 |  | 1.396 | 1.394 | 1.393 | 1.394 | ± 0.001 |  |
| *L. salivarius* strain PUPro4 | 1.293 | 1.293 | 1.296 | 1.294 | ± 0.001 |  | 1.287 | 1.286 | 1.281 | 1.284 | ± 0.003 |  |

**Supplementary data Table 9:** Resistance of present study isolates against *Bacillus subtilis* estimated through antimicrobial resistance assay

**Supplementary data Table 10:** Resistance of present study isolates against *Bacillus proteus* estimated through antimicrobial resistance assay

| Isolates | *Bacillus proteus* | | | | | | | | | | | |
| --- | --- | --- | --- | --- | --- | --- | --- | --- | --- | --- | --- | --- |
|  | **OD OF CONTROL AT (600nm)** | | | | | | **OD OF ISOLATES & PATHOGEN AT (600nm)** | | | | | |
|  | **I** | **II** | **III** | **Mean** | **Standard Deviation** | **p-value** | **I** | **II** | **III** | **Mean** | **Standard Deviation** | **p-value** |
| *L. antri* strain PUPro1 | 1.02 | 1.099 | 1.022 | 1.047 | ± 0.045 | < 0.05 | 1.019 | 1.016 | 1.009 | 1.014 | ± 0.005 | < 0.05 |
| *L. delbrueckii* strain PUPro2 | 1.016 | 1.017 | 1.014 | 1.015 | ± 0.001 |  | 1.008 | 1.007 | 1.006 | 1.007 | ± 0.001 |  |
| *L. casei* strain PUPro3 | 1.043 | 1.042 | 1.044 | 1.043 | ± 0.001 |  | 1.03 | 1.033 | 1.036 | 1.033 | ± 0.003 |  |
| *L. salivarius* strain PUPro4 | 0.983 | 0.983 | 0.989 | 0.985 | ± 0.003 |  | 0.972 | 0.973 | 0.969 | 0.971 | ± 0.002 |  |

**Supplementary data Table 11:** Resistance of present study isolates against *Staphylococcus aureus* estimated through antimicrobial resistance assay

| Isolates | *Staphylococcus aureus* | | | | | | | | | | | |
| --- | --- | --- | --- | --- | --- | --- | --- | --- | --- | --- | --- | --- |
|  | **OD OF CONTROL AT (600nm)** | | | | | | **OD OF ISOLATES & PATHOGEN AT (600nm)** | | | | | |
|  | **I** | **II** | **III** | **Mean** | **Standard Deviation** | **p-value** | **I** | **II** | **III** | **Mean** | **Standard Deviation** | **p-value** |
| *L. antri* strain PUPro1 | 0.201 | 1.202 | 1.199 | 0.867 | ± 0.577 | < 0.05 | 1.189 | 1.186 | 1.184 | 1.186 | ± 0.161 | < 0.05 |
| *L. delbrueckii* strain PUPro2 | 0.947 | 0.939 | 0.946 | 0.944 | ± 0.004 |  | 0.92 | 0.924 | 0.917 | 0.92 | ± 0.172 |  |
| *L. casei* strain PUPro3 | 0.897 | 0.886 | 0.893 | 0.892 | ± 0.005 |  | 0.86 | 0.861 | 0.864 | 0.861 | ± 0.158 |  |
| *L. salivarius* strain PUPro4 | 1.349 | 1.347 | 1.351 | 1.349 | ± 0.002 |  | 1.321 | 1.324 | 1.32 | 1.321 | ± 0.073 |  |

| Isolates | (a) AMOXILIN | | | | | | |  |  | Isolates | (b) AZITHROMYCIN | | | | | |
| --- | --- | --- | --- | --- | --- | --- | --- | --- | --- | --- | --- | --- | --- | --- | --- | --- |
|  | **5**  **µl** | **10**  **µl** | **15**  **µl** | **Mean** | | **Standard Deviation** | **p-value** |  |  |  | **5**  **µl** | **10µl** | **15µl** | **Mean** | **Standard Deviation** | **p-value** |
| *L. antri* strain PUPro1 | 29 | 32 | 33 | 31.33 | | ± 2.08 | < 0.05 |  |  | ***L. antri* strain PUPro1** | 25 | 29 | 32 | 28.67 | ± 3.511 | < 0.05 |
| *L. delbrueckii* strain PUPro2 | 31 | 33 | 33 | 32.33 | | ± 1.15 |  |  |  | ***L. delbrueckii* strain PUPro2** | 21 | 24 | 26 | 23.67 | ± 2.516 |  |
| *L. casei* strain PUPro3 | 32 | 35 | 36 | 34.33 | | ± 2.08 |  |  |  | ***L. casei* strain PUPro3** | 17 | 19 | 23 | 19.67 | ± 3.055 |  |
| *L. salivarius* strain PUPro4 | 20 | 28 | 29 | 25.67 | | ± 4.93 |  |  |  | ***L. salivarius* strain PUPro4** | 22 | 26 | 29 | 25.67 | ± 3.511 |  |
|  |  |  |  |  | |  | |  |  |  |  |  |  |  |  | |
|  |  |  |  |  | |  | |  |  |  |  |  |  |  |  | |
| Isolates | **(c) CEFADROXIL** | | | | | | |  |  | **Isolate** | **(d) VELOSEF** | | | | | |
|  | **5**  **µl** | **10**  **µl** | **15**  **µl** | **Mean** | | **Standard Deviation** | **p-value** |  |  |  | **5µl** | **10µl** | **15µl** | **Mean** | **Standard Deviation** | **p-value** |
| *L. antri* strain PUPro1 | 20 | 22 | 24 | 22 | | ± 2 | < 0.05 |  |  | ***L. antri* strain PUPro1** | 28 | 29 | 32 | 29.67 | ± 2.081 | <0.05 |
| *L. delbrueckii* strain PUPro2 | 19 | 22 | 26 | 22.33 | | ± 3.511 |  |  |  | ***L. delbrueckii* strain PUPro2** | 19 | 21 | 28 | 22.67 | ± 4.725 |  |
| *L. casei* strain PUPro3 | 22 | 24 | 27 | 24.33 | | ± 2.516 |  |  |  | ***L. casei* strain PUPro3** | 17 | 19 | 23 | 19.67 | ± 3.055 |  |
| *L. salivarius* strain PUPro4 | 17 | 18 | 17 | 17.33 | | ± 0.577 |  |  |  | ***L. salivarius* strain PUPro4** | 32 | 34 | 35 | 33.67 | ± 1.527 |  |
|  |  |  |  |  | |  | |  |  |  |  |  |  |  |  | |
|  |  |  |  |  | |  | |  |  |  |  |  |  |  |  | |
| Isolates | **(e) KANAMYCIN** | | | | | | |  |  | **Isolates** | **(f) AUGMENTIN** | | | | | |
|  | **5µl** | **10µl** | **15µl** | **Mean** | **Standard Deviation** | | **p-value** |  |  |  | **5µl** | **10µl** | **15µl** | **Mean** | **Standard Deviation** | **p-value** |
| *L. antri* strain PUPro1 | 23 | 28 | 29 | 26.67 | ± 3.214 | | < 0.05 |  |  | ***L. antri* strain PUPro1** | 26 | 28 | 32 | 28.67 | ± 3.055 | <0.05 |
| *L. delbrueckii* strain PUPro2 | 20 | 22 | 26 | 22.67 | ± 3.055 | |  |  |  | ***L. delbrueckii* strain PUPro2** | 29 | 31 | 33 | 31 | ± 2 |  |
| *L. casei* strain PUPro3 | 28 | 29 | 34 | 30.33 | ± 3.214 | |  |  |  | ***L. casei* strain PUPro3** | 32 | 34 | 35 | 33.67 | ± 1.527 |  |
| *L. salivarius* strain PUPro4 | 20 | 31 | 36 | 29 | ± 8.185 | |  |  |  | ***L. salivarius* strain PUPro4** | 37 | 39 | 42 | 39.33 | ± 2.516 |  |

**Supplementary data Table 12:** Zones of inhibition (mm) exhibited by present study bacteria against (a) amoxilin (b) azithromycin (c) cefadroxil (d) velosef (e) kanamycin (f) augmentin

**Supplementary data Table 13:** Hemolytic activities of probiotic bacteria

| Isolate | Type of Haemolysis |
| --- | --- |
| *L. antri* strain PUPro1 | ϒ-haemolysis |
| *L. delbrueckii* strain PUPro2 | ϒ-haemolysis |
| *L. casei* strain PUPro3 | ϒ-haemolysis |
| *L. salivarius* strain PUPro4 | ϒ-haemolysis |
